# GLP-1R agonists demonstrate potential to treat Wolfram syndrome in human preclinical models

**DOI:** 10.1007/s00125-023-05905-8

**Published:** 2023-03-30

**Authors:** Vyron Gorgogietas, Bahareh Rajaei, Chae Heeyoung, Bruno J. Santacreu, Sandra Marín-Cañas, Paraskevi Salpea, Toshiaki Sawatani, Anyishai Musuaya, María N. Arroyo, Cristina Moreno-Castro, Khadija Benabdallah, Celine Demarez, Sanna Toivonen, Cristina Cosentino, Nathalie Pachera, Maria Lytrivi, Ying Cai, Lode Carnel, Cris Brown, Fumihiko Urano, Piero Marchetti, Patrick Gilon, Decio L. Eizirik, Miriam Cnop, Mariana Igoillo-Esteve

**Affiliations:** 1 ULB Center for Diabetes Research, Université Libre de Bruxelles, Brussels, Belgium; 2 Institut de Recherche Expérimental et Clinique, Pôle d’Endocrinologie, Diabète et Nutrition, Université Catholique de Louvain, Bruxelles, Belgique; 3 Division of Endocrinology, Erasmus Hospital, Université Libre de Bruxelles, Brussels, Belgium; 4 Eye Hope Foundation, Damme, Belgium; 5 Department of Medicine, Washington University School of Medicine in St Louis, St Louis, MO USA; 6 Department of Clinical and Experimental Medicine, AOUP Cisanello University Hospital, University of Pisa, Pisa, Italy

**Keywords:** GLP-1R agonists, Human pancreatic beta cells, iPSC-derived beta cells, iPSC-derived neurons, Wolfram syndrome

## Abstract

**Aims/hypothesis:**

Wolfram syndrome is a rare autosomal recessive disorder caused by pathogenic variants in the *WFS1* gene. It is characterised by insulin-dependent diabetes mellitus, optic nerve atrophy, diabetes insipidus, hearing loss and neurodegeneration. Considering the unmet treatment need for this orphan disease, this study aimed to evaluate the therapeutic potential of glucagon-like peptide 1 receptor (GLP-1R) agonists under wolframin (WFS1) deficiency with a particular focus on human beta cells and neurons.

**Methods:**

The effect of the GLP-1R agonists dulaglutide and exenatide was examined in *Wfs1* knockout mice and in an array of human preclinical models of Wolfram syndrome, including WFS1-deficient human beta cells, human induced pluripotent stem cell (iPSC)-derived beta-like cells and neurons from control individuals and individuals affected by Wolfram syndrome*,* and humanised mice.

**Results:**

Our study shows that the long-lasting GLP-1R agonist dulaglutide reverses impaired glucose tolerance in WFS1-deficient mice, and that exenatide and dulaglutide improve beta cell function and prevent apoptosis in different human WFS1-deficient models including iPSC-derived beta cells from people with Wolfram syndrome. Exenatide improved mitochondrial function, reduced oxidative stress and prevented apoptosis in Wolfram syndrome iPSC-derived neural precursors and cerebellar neurons.

**Conclusions/interpretation:**

Our study provides novel evidence for the beneficial effect of GLP-1R agonists on WFS1-deficient human pancreatic beta cells and neurons, suggesting that these drugs may be considered as a treatment for individuals with Wolfram syndrome.

**Graphical abstract:**

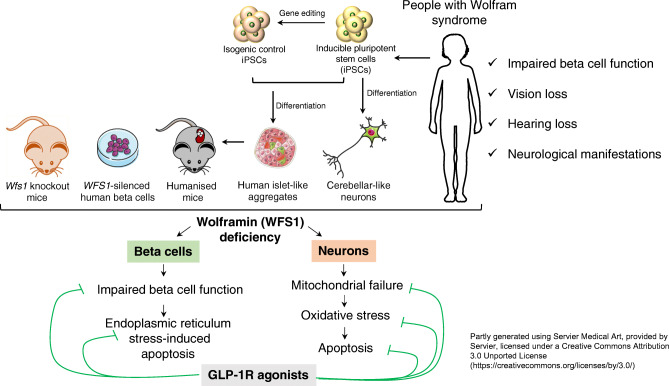

**Supplementary Information:**

The online version of this article (10.1007/s00125-023-05905-8) contains peer-reviewed but unedited supplementary material.

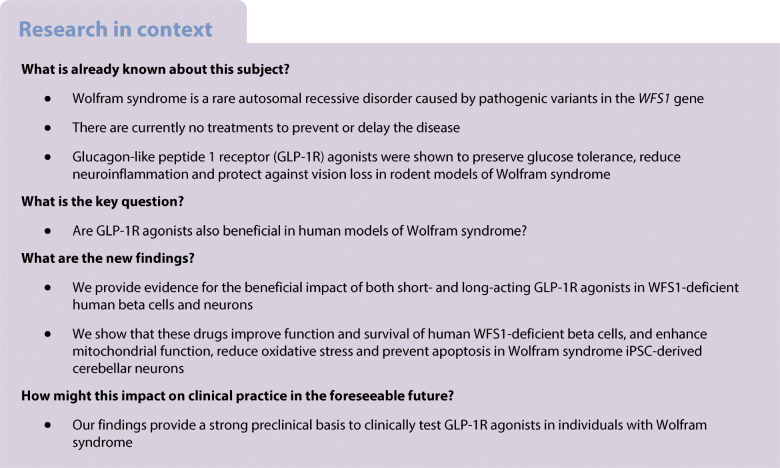



## Introduction

Wolfram syndrome is a rare autosomal recessive disorder with a prevalence of one in 700,000 individuals [1, 2]. It is caused by mutations in the *WFS1* gene which encodes for the endoplasmic reticulum (ER) transmembrane protein WFS1, also called wolframin [3]. In the first two decades of life, individuals affected by Wolfram syndrome develop insulin-dependent diabetes mellitus, optic nerve atrophy, diabetes insipidus and hearing loss [4]. People with Wolfram syndrome can also develop cerebellar ataxia, gait and balance abnormalities, memory loss and psychiatric manifestations [5]. There are currently no treatments to prevent or delay the disease.

The ER is an essential organelle for pancreatic beta cells and neurons. WFS1 deficiency perturbs ER Ca^2+^ homeostasis and causes ER stress [2, 6–8], defined as an imbalance between the protein load in the ER and the organelle’s functional capacity. The ER stress response is an adaptive process aiming to restore ER homeostasis. When prolonged, it can lead to cell dysfunction and death [9]. In beta cells, WFS1 deficiency impairs insulin synthesis and secretion and induces apoptosis [4, 6, 7]. In brain, it results in delayed neuronal development and impaired neuronal survival [10]. WFS1 deficiency also causes mitochondrial dysfunction because of altered ER–mitochondria interactions and Ca^2+^ exchange [10, 11], and impairs granule acidification in beta cells and neurons, necessary for proper insulin and neurotransmitter release [4, 12].

Glucagon-like peptide 1 (GLP-1) is an incretin hormone produced by enteroendocrine L cells in ileum and colon and in brainstem neurons [13]. It binds its G-protein-coupled receptor (glucagon-like peptide 1 receptor, GLP-1R), stimulates cAMP formation and thereby enhances insulin secretion and beta cell survival, including in conditions of ER stress [14, 15]. GLP-1R agonists with prolonged half-life (generated by amino acid substitutions and linkage to a fatty acid, the Fc region of IgG4 or albumin [16]) have been developed to treat type 2 diabetes [17]. These modifications result in half-lives of 2.4 h for exenatide, 12 h for liraglutide and 120 h for dulaglutide [16]. GLP-1Rs are also expressed in neurons, astrocytes, microglia and retinal cells [18, 19]. Some GLP-1R agonists cross the blood–brain barrier [20] and confer neuroprotection by enhancing neuronal stem cell activation and synaptic plasticity and cognition, and exerting anti-inflammatory effects [19]. GLP-1R agonists have hence been examined as a potential therapy for diabetes, neurodegeneration and vision loss in Wolfram syndrome [21–26]. Acute exenatide injection in *Wfs1* knockout (KO) mice enhances insulin secretion [23], while prolonged exenatide and liraglutide administration in young WFS1-deficient mice and rats improves glucose-stimulated insulin secretion and protects against vision loss [21, 22, 25]. In older WFS1-deficient rats, liraglutide reduces neuroinflammation, delays optic nerve atrophy and improves visual acuity [24, 26].

The translational success of rodent studies to human disease is difficult to predict, as mice and rats often fail to recapitulate key pathophysiological features of human disease and treatment responses [27, 28]. Therefore, considering the unmet need of treatment for this orphan disease, we set out to test the therapeutic potential of GLP-1R agonists not only in WFS1-deficient mice, but also in human WFS1-deficient beta cells and neurons, and in a humanised mouse model of Wolfram syndrome.

## Methods

For detailed methods and ethics please refer to the electronic supplementary material (ESM) Methods.

### Ethical approvals

All mouse experiments were approved by the Commission d’Ethique et du Bien Être Animal (CEBEA), Faculty of Medicine, Université Libre de Bruxelles.

Isolation and use of human islets, skin biopsies, fibroblast reprogramming and induced pluripotent stem cell (iPSC) differentiation were approved by the Human Research Ethics Committees of the Universities of Pisa, Alberta and Barcelona; the Columbia Institutional Review Board and Embryonic Stem Cell Research Oversight Committee; and the Ethical Committee of Erasmus Hospital, Université Libre de Bruxelles, as appropriate.

### Mice

*Wfs1* KO mice (*Wfs1*^tm1^Koks) with a homozygous exon 8 deletion on 129S6 background were kindly provided by S. Kõks, University of Tartu, Estonia [29]. Wild-type (WT) littermates were used as controls.

### GLP-1R agonist administration and in vivo metabolic studies

Male and female *Wfs1* KO and WT littermate mice received dulaglutide (1 mg/kg every 4 days) or vehicle (saline solution [154 mmol/l NaCl]) by i.p. injection as previously described [30]. The treatment was initiated at 4, 7 or 21 weeks of age. At 48 h after dulaglutide or saline injection, IPGTTs and ITTs were performed. Mice were killed by cervical dislocation, and pancreases were harvested and fixed in 4% formalin for histological analysis.

### EndoC-βH1, human islets and iPSC lines

The human beta cell line EndoC-βH1 [31], kindly provided by R. Scharfmann (Cochin Institute, Paris, France), was cultured as described [31].

Human islets from nondiabetic organ donors were isolated in Pisa, Italy; Edmonton, Canada; or Barcelona, Spain, and shipped to Brussels. The human islet checklist is provided in the ESM. EndoC-βH1 and dispersed islet cells were transfected with control siRNA (siCT) that does not interfere with cell function or gene expression [32] or with two siRNAs targeting human *WFS1* (ESM Tables 1, 2).

iPSC lines (Wolf-2010-07.1, Wolf-2010-11.1, Wolf-2011-13.2 and Wolf-2010-9.4) [33, 34] from four individuals with Wolfram syndrome and one isogenic control line (Wolf-9.4-Corr-2G6.1), generated by CRISPR-Cas9 editing of Wolf-2010-9.4, were used for the differentiations. iPSCs were differentiated into beta-like cell aggregates using a seven-stage protocol [35–38]. *WFS1* variants and patient characteristics are shown in ESM Table 3. Differentiation efficiency was assessed by real-time PCR and immunofluorescence using primers listed in ESM Tables 4, 5, and antibodies listed in ESM Table 6.

iPSCs were differentiated into cerebellar neuron-like cells as described [39], with slight modifications. Neural precursor cells (NPCs) and a mixture of immature cerebellar neurons, obtained at the end of the differentiation, were used for the experiments. Medium composition and plating cell densities are available in ESM Tables 7–11.

### Cell treatments and cell death assays

EndoC-βH1 cells, dispersed human islets, iPSC-derived beta cells, NPCs and cerebellar neurons were exposed to the ER stressor tunicamycin (5 μg/ml), thapsigargin (1 μmol/l) or brefeldin A (0.05 μg/ml), alone or combined with exenatide (50 nmol/l in beta cells, 500 nmol/l in neurons), dulaglutide (50 nmol/l) or forskolin (20 μmol/l). EndoC-βH1 cells were pretreated with exenatide, dulaglutide or forskolin for 2 h, and other cell types for 24 h.

Cell death was assessed by fluorescence microscopy counting after staining with DNA binding dyes Hoechst 33342 and propidium iodide. Early and late apoptosis was assessed by RealTime-Glo Annexin V apoptosis and necrosis assay (Promega, USA) [35].

### iPSC-derived beta cell transplantation into immunodeficient mice

iPSC-derived beta cell aggregates were transplanted into 5–7-week-old male *Rag2* KO mice. IPGTT was performed 7 and 14 weeks after transplantation, and plasma C-peptide was measured by ELISA [38]. Fourteen weeks after transplantation, mice were allocated to i.p. dulaglutide (1 mg/kg every 4 days) or vehicle injection for 12 weeks by simple randomisation. Mice were killed by cervical dislocation and graft function was assessed by in situ kidney perifusion. To assess grafted iPSC-derived beta cell function, the kidney was perfused in situ at 37°C in a single-pass circuit through the renal artery [38].

### Immunofluorescence, western blot, real-time PCR and reactive oxygen species detection

For immunostaining, cells were fixed in 4% formaldehyde, permeabilised, blocked, incubated with primary and fluorescent secondary antibodies, and mounted in Vectashield Vibrance Antifade Mounting Medium with DAPI (Vector Laboratories, USA).

Proteins were resolved in SDS-PAGE, transferred to nitrocellulose membranes and blotted with specific primary and horseradish-conjugated secondary antibodies (ESM Table 6).

mRNA was isolated and reverse transcribed as described [36]. Gene expression was assessed on a MyiQ2 Single-Color Real-Time PCR System (Bio-Rad, USA). *GAPDH* and/or beta-actin (*ACTB*) were used as reference genes.

Intracellular reactive oxygen species were detected using the fluorescent probe hydroxyphenyl fluorescein (HPF; Invitrogen, USA).

### Mitochondrial respiration

Mitochondrial function was assessed by measuring oxygen consumption rate (OCR) in a Seahorse XFp Extracellular Flux Analyzer (Agilent, USA). Before the assay, dispersed iPSC-derived beta cells, NPCs and cerebellar neurons were exposed or not to exenatide or forskolin for 72 h. OCR data were normalised to the last basal reading in each sample.

### Statistical analysis

Data are shown using bars or floating bars (minimum to maximum), with a horizontal line representing the median. Individual data points represent independent experiments or individual mice. Comparisons between groups were performed by two-way ANOVA (or mixed effects analysis in the case of missing values) or one-way ANOVA followed by Tukey’s, Sidak’s or Dunn’s test or the two-stage step-up method of Benjamini, Krieger and Yekutieli correction for multiple comparisons (as recommended by GraphPad Prism 9, https://www.graphpad.com/scientific-software/prism/). A *p* value <0.05 was considered statistically significant.

## Results

### Dulaglutide preserves glucose tolerance in young WFS1-deficient mice

**Fig. 1:**
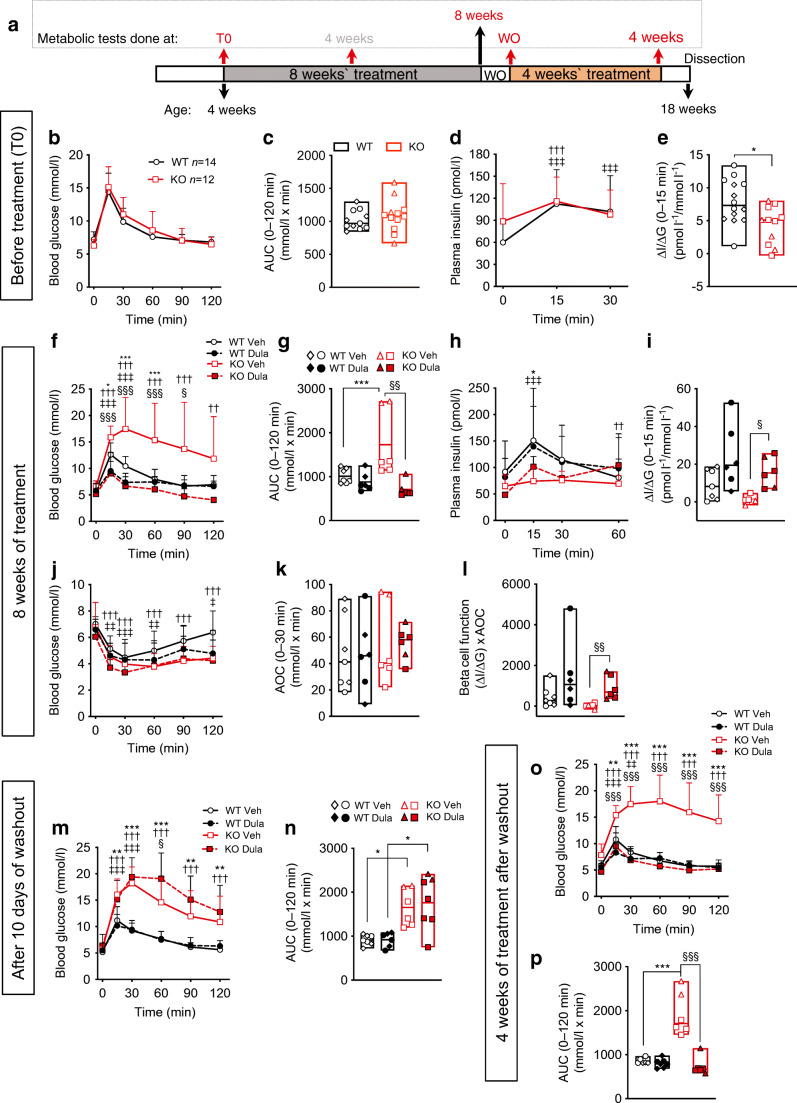
Dulaglutide treatment prevents glucose intolerance in WFS1-deficient mice. (**a**) Schematic representation of the experiment. Four-week-old *Wfs1* KO and WT littermates were treated for 8 weeks with saline (Veh, *n*=6–7) or dulaglutide (1 mg/kg every 4 days, Dula, *n*=6–7). After 8 weeks, treatment was suspended for 10 days (washout, WO), and then resumed for 4 more weeks. Timing of metabolic tests (IPGTT and ITT) is indicated. (**b**, **f**, **m**, **o**) IPGTT glucose levels, (**c**, **g**, **n**, **p**) AUC and (**d**, **h**) insulin levels. (**e**, **i**) Insulinogenic index, (**j**) ITT glucose levels, (**k**) insulin sensitivity calculated as ITT area over the curve (AOC) and (**l**) beta cell function. Results are expressed as mean and SD. In the bars, circles and squares represent individual female mice, triangles and diamonds male mice. Extremities of floating bars are maximal and minimal values; horizontal line shows median. *p<0.05, **p<0.01, ***p<0.001 KO Veh vs WT Veh; ^††^*p*<0.01, ^†††^*p*<0.01 other time points vs T0 in KO Veh; ^‡^*p*<0.05, ^‡‡^*p*<0.01, ^‡‡‡^*p*<0.001 other time points vs T0 in WT Veh; ^§^*p*<0.05, ^§§^*p*<0.01, ^§§§^*p*<0.001 Dula vs Veh in KO, by two-way or one-way ANOVA (as suitable) followed by Sidak’s or Dunn’s correction for multiple comparisons. ΔI/ΔG, incremental insulin over incremental glucose; T0, time zero (treatment initiation)

We administered the long-lasting GLP-1 analogue dulaglutide to 4-week-old *Wfs*1 KO and WT mice for 8 weeks, interrupted treatment for 10 days (washout) and then resumed it for 4 more weeks. Metabolic tests were performed at the time points specified in Fig. 1a. The IPGTT before treatment initiation showed that glucose tolerance (Fig. 1b,c) and insulin levels (Fig. 1d) were similar between genotypes at age 4 weeks. *Wfs1* KO mice had slightly lower acute insulin response to glucose (Fig. 1e), and a non-significant trend for higher fasting insulin levels (*p*=0.23). After 4 weeks, vehicle-treated *Wfs1* KO mice had impaired glucose tolerance with respect to WT mice (ESM Fig. 1a,b) and, as previously reported [40], male *Wfs1* KO mice had higher glucose levels than females (ESM Fig. 1b). These differences were also present after 8 weeks of treatment (Fig. 1f,g). Saline-treated *Wfs1* KO mice tended to have reduced insulin levels and acute insulin response to glucose when compared with WT mice (Fig. 1h,i and ESM Fig. 1c,d). Four week dulaglutide administration normalised glucose tolerance in *Wfs1* KO mice (ESM Fig. 1a,b) and enhanced acute insulin response to glucose (ESM Fig. 1d). After 8 weeks on dulaglutide, WFS1-deficient mice were still normoglycaemic (Fig. 1f,g) and had improved insulin levels, acute insulin response to glucose and beta cell function (Fig. 1h,i,l), showing durable beneficial effects of the treatment. Insulin sensitivity, assessed by ITT, was similar between *Wfs1* KO and WT mice (Fig. 1j,k). In WT mice, dulaglutide administration resulted in a non-significant improvement in glucose tolerance (*p*=0.47) and insulin secretion (*p*=0.11) at 4 weeks (ESM Fig. 1a–c) but this effect was lost after 8 weeks of treatment (Fig. 1f–h). When the treatment was discontinued for 10 days, its protective effect was lost (Fig. 1m,n): *Wfs1* KO mice became as glucose intolerant as saline-injected mice, suggesting that the GLP-1R agonists essentially have a transient secretagogue effect which is lost upon drug removal. This impairment in glucose tolerance was fully reversed when dulaglutide was resumed for 4 more weeks (Fig. 1o,p). Insulin and glucagon immunostaining of pancreas harvested at the end of the treatment showed that islets from *Wfs1* KO mice had reduced beta and enhanced alpha cell proportions compared with WT islets, and no difference in islet size (ESM Fig. 2). Dulaglutide had no discernible impact on islet size and composition.

**Fig. 2:**
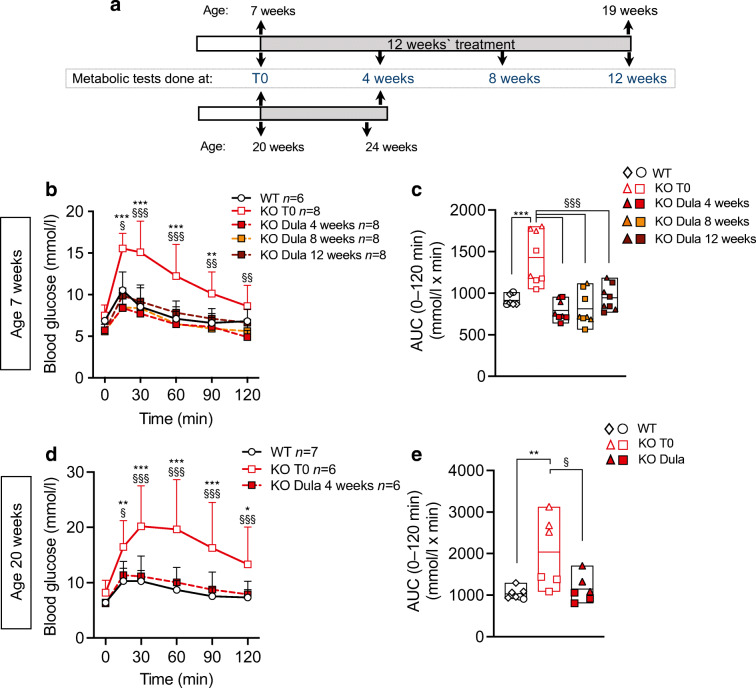
Dulaglutide treatment reverses diabetes in WFS1-deficient mice. (**a**) Schematic representation of the experiment. Seven-week-old (*n*=8) (**b**, **c**) or 20-week-old (*n*=6) (**d**, **e**) *Wfs1* KO mice were treated for 4–12 weeks with dulaglutide (1 mg/kg every 4 days) and compared with age-matched WT littermates. (**b**, **d**) IPGTT glucose levels and (**c**, **e**) AUC of WT mice and dulaglutide-injected *Wfs1* KO mice at baseline (T0) and after 4, 8 and 12 weeks of treatment. Results are expressed as mean and SD. In the bars circles and squares represent individual female mice, triangles and diamonds male mice. Extremities of floating bars are maximal and minimal values; horizontal line shows median. *p<0.05, **p<0.005, ***p<0.001 KO T0 vs WT ; ^§^*p*<0.05, ^§§^*p*<0.05, ^§§§^*p*<0.001 Dula vs KO T0, by two-way or one-way ANOVA (as suitable) followed by Sidak’s or Dunn’s correction for multiple comparisons. Dula, dulaglutide; T0, time zero (treatment initiation)

To assess whether dulaglutide could indeed reverse impaired glucose tolerance or diabetes (Fig. 2a), we treated 7- and 20-week-old *Wfs1* KO mice for 4–12 weeks (Fig. 2b–e). Four week dulaglutide administration normalised glucose tolerance of WFS1-deficient mice of both ages (Fig. 2b–e) and this positive effect was maintained over a 12 week treatment course (Fig. 2c). Altogether, these results indicate that dulaglutide has remarkable beneficial effects on glucose tolerance of WFS1-deficient mice, in preventive as well as curative settings, without, however, being disease-modifying, as indicated by the loss of glucose tolerance upon washout and absence of effect on beta cell mass.

### Exenatide protects WFS1-deficient human beta cells from ER stress-induced apoptosis

**Fig. 3:**
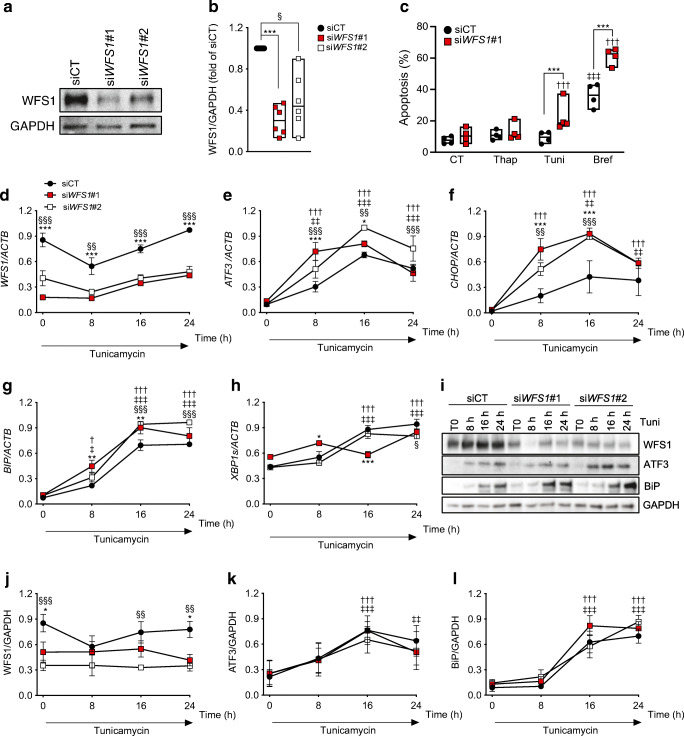
*WFS1* silencing sensitises human beta cells to ER stress. *WFS1* was silenced or not (siCT) in EndoC-βH1 cells using two siRNAs (si*WFS1*#1 and #2). (**a**) Representative western blot and (**b**) densitometric quantification of WFS1 knockdown 72 h after transfection. GAPDH was used as a control for protein loading. (**c**) Apoptosis assessed by Hoechst 33342/propidium iodide staining in control and *WFS1*-silenced EndoC-βH1 cells exposed or not for 24 h to thapsigargin, tunicamycin or brefeldin. Data points represent independent experiments. Extremities of floating bars are maximal and minimal values; horizontal line shows median. (**d**–**l**) Time course of tunicamycin exposure in EndoC-βH1 cells silenced for *WFS1* for 48 h. *WFS1*, *ATF3*, *CHOP*, *BIP* and *XBP1s* mRNA expression was measured by real-time PCR (**d**–**h**) and normalised to reference gene *ACTB*. WFS1, ATF3 and BiP expression was examined by western blot and normalised to the reference protein GAPDH (**i**–**l**). Results are means ± SEM of *n*=3 (real-time PCR) or *n*=4 (western blots) independent experiments, and are expressed as fold of the highest value in each experiment. *p<0.05, **p<0.01, ***p<0.001 si*WFS1*#1 vs siCT*,* §*p*<0.05*,* §§*p<*0.01*,* §§§*p<*0.001 *siWFS1*#2 vs siCT; ^†^*p*<0.05, ^††^*p*<0.01, ^†††^*p*<0.001 treated vs CT or vs time 0 h in WFS1-deficient cells; ^‡^*p*<0.05, ^‡‡^*p*<0.01, ^‡‡‡^*p*<0.001 treated vs CT or vs time 0 h in control cells. Data were analysed by one-way or two-way ANOVA (as suitable), followed by Sidak’s or Dunnett’s test for multiple comparisons. Bref, brefeldin; CT, control untreated; Thap, thapsigargin; Tuni, tunicamycin

We next investigated the effect of GLP-1R agonists in WFS1-deficient human beta cells. Exenatide and the cAMP inducer forskolin were previously shown to protect beta cells from ER stress-induced apoptosis [15, 41]. Two siRNAs targeting *WFS1* induced a 50–70% decrease in WFS1 protein expression in EndoC-βH1 cells (Fig. 3a,b), marginally affected basal beta cell viability and enhanced apoptosis induced by the ER stressors tunicamycin and brefeldin but not thapsigargin (Fig. 3c). In time course experiments of tunicamycin exposure, WFS1 deficiency enhanced ER stress at early time points (8–16 h), with increased mRNA expression of the transcription factor *ATF3*, proapoptotic transcription factor *CHOP* (also known as *DDIT3*) and ER chaperone *BIP* (also known as *HSPA5*) but no consistent changes in spliced *XBP1* (Fig. 3d–h). At the protein level, WFS1-deficient cells showed a non-significant trend for increased ER chaperone BiP (BiP) expression (*p*=0.23) (Fig. 3i–l).

**Fig. 4:**
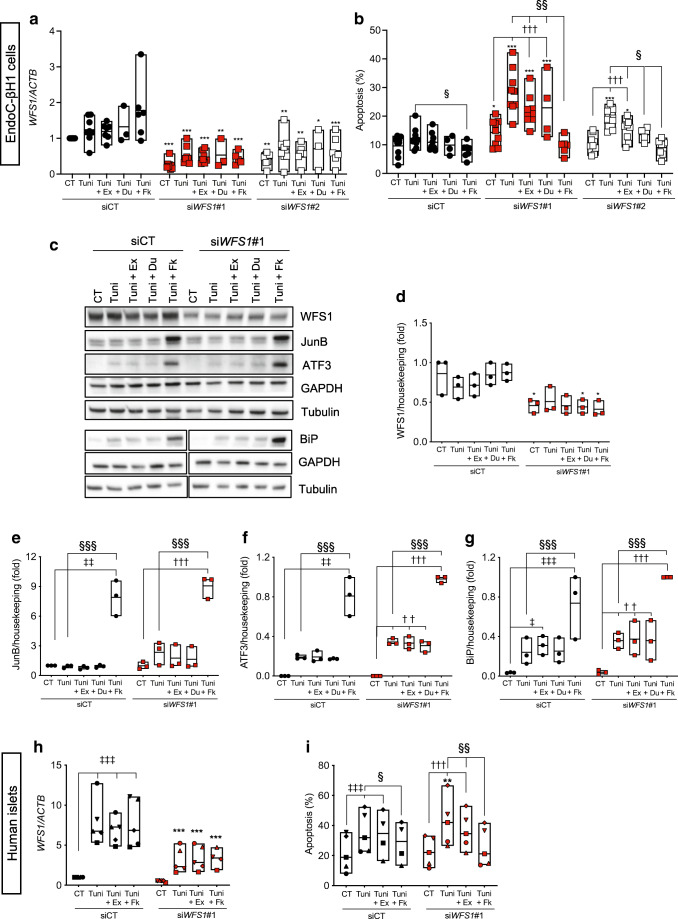
Exenatide protects human beta cells from ER stress-induced apoptosis. *WFS1* was silenced in EndoC-βH1 cells (**a**–**g**) or dispersed human islets (**h**–**i**) using siRNAs (si*WFS1*#1 and #2). At 24 h after transfection, the cells were pretreated or not for 2 h or 24 h, respectively, with exenatide (50 nmol/l), dulaglutide (50 mmol/l) or forskolin (20 μmol/l), and then exposed or not (CT) for 24 (**a**, **b**), 16 (**c**–**g**) or 48 h (**h**, **i**), respectively, to tunicamycin alone or in combination with exenatide, forskolin or dulaglutide. (**a**, **h**) *WFS1* mRNA expression by real-time PCR. (**b**, **i**) Apoptosis evaluated by Hoechst 33342/propidium iodide staining. (**c**–**g**) Western blot data. WFS1, JunB, ATF3 and BiP protein expression was normalised to the geometric mean of the reference proteins tubulin and GAPDH, and expressed as fold of the highest value in each experiment. Data points represent independent experiments. Extremities of the floating bars are maximal and minimal values; horizontal line shows median. *p<0.05, **p<0.01, ***p<0.001 si*WFS1*#1 or *siWFS*#2 vs the same treatment in siCT; ^††^*p*<0.01, ^†††^*p*<0.001 treated vs CT in *WFS1*-silenced cells; ^‡^*p*<0.05, ^‡‡^*p*<0.01, ^‡‡‡^*p*<0.001 treated vs CT in control cells; ^§^*p*<0.05, ^§§^*p*<0.05, ^§§§^*p*<0.001 Tuni+Ex or Tuni+Fk vs Tuni, by one-way ANOVA followed by Sidak’s or Tukey’s test for multiple comparisons. CT, control (vehicle); Du, dulaglutide; Ex, exenatide; Fk, forskolin; Tuni, tunicamycin

Exenatide and dulaglutide attenuated cell death in *WFS1*-silenced EndoC-βH1 cells (Fig. 4a,b), while forskolin conferred full protection (Fig. 4b). Exenatide and dulaglutide did not reduce markers of ER stress (ESM Fig. 3). Forskolin increased expression of the antiapoptotic transcription factor JunB (JunB), its downstream target activating transcription factor 3 (ATF3) and BiP (Fig. 4c–g), suggesting that the protection conferred by this drug results from the combined expression of these proteins previously shown to protect ER-stressed beta cells [15, 42]. Exenatide or dulaglutide did not alter JunB/ATF3 or BiP protein expression, but the former induced *ATF3* mRNA expression (ESM Fig. 3i). Exenatide and forskolin also significantly reduced apoptosis in *WFS1*-silenced primary human islet cells (Fig. 4h,i).

### Exenatide protects Wolfram syndrome iPSC-derived beta cells from ER stress

We next turned to more disease-relevant models, namely iPSC-derived beta cells and cerebellar neurons. Of the Wolfram syndrome (Wolf 2010-07.1, Wolf-2010-11.1, Wolf-2011-13.2 and Wolf-2010-9.4) [33, 34] and isogenic control (Wolf-9.4-Corr-2G6.1) iPSC lines, three have been validated previously as genuine iPSCs [33, 34]. We validated the other two (Wolf 2010-07.1 and Wolf 9.4-Corr-2G6.1), showing that they had normal karyotype, expressed pluripotency markers and differentiated into the three germ layers (ESM Fig. 4).

**Fig. 5:**
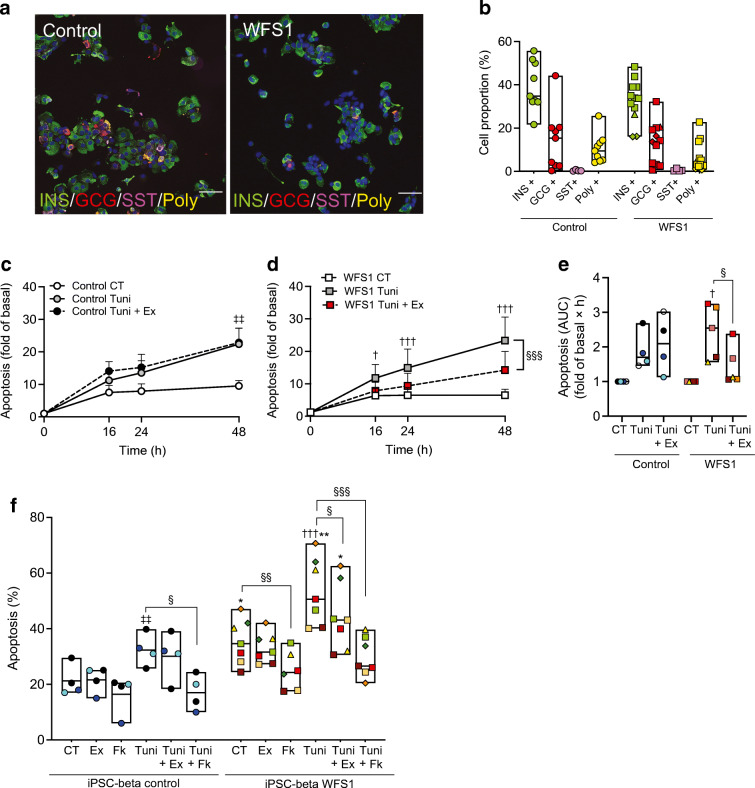
Exenatide protects iPSC-derived Wolfram syndrome beta-like cells from ER stress-induced apoptosis. Wolfram syndrome iPSCs (Wolf-2010-11.1, Wolf-2011-13.2 and Wolf-2010-9.4) and the isogenic control line (Wolf-9.4-Corr-2G6.1) were differentiated into beta-like cells. (**a**) Representative images and (**b**) quantification of dispersed stage 7 aggregate cells stained for insulin (green), glucagon (red) and somatostatin (pink). Polyhormonal cells (Poly) are coloured yellow. Nuclei were stained with DAPI. Scale bar, 20 μm. Whole (**c**–**e**) or dispersed (**f**) aggregates were pretreated or not for 24 h with exenatide (50 nmol/l) or forskolin (20 μmol/l) and then exposed or not (CT) for 48 h to tunicamycin (Tuni), alone or combined with exenatide (Ex) or forskolin (Fk). (**c**, **d**) Time course of early and late apoptosis in whole control (**c**) or WFS1 (**d**) aggregates assessed by RealTime-Glo Annexin V. Results are expressed as fold of basal apoptosis in each condition. (**e**) AUC of (**c**) and (**d**). (**f**) Apoptosis determined by Hoechst 33342/propidium iodide staining in dispersed aggregates. Circles represent differentiations of Wolf-9.4-Corr-2G6.1, triangles Wolf-2010-11.1, diamonds Wolf-2011-13.2 and squares Wolf-2010-9.4. Extremities of floating bars are maximal and minimal values; horizontal line shows median. *p<0.05, **p<0.01 WFS1 vs control; ^†^*p*<0.05, ^†††^*p*<0.001 Tuni vs CT in WFS1 cells; ^‡‡^*p*<0.01 Tuni vs CT in control cells; ^§^*p*<0.05, ^§§^*p*<0.01, ^§§§^*p*<0.001 Fk vs CT, Tuni+Ex or Tuni+Fk vs Tuni, by one-way or two-way ANOVA or mixed effects analysis followed by the two-stage step-up method of Benjamini, Krieger and Yekutieli or Tukey’s test for multiple comparisons. CT, control (vehicle); GCG, glucagon; INS, insulin; SST, somatostatin

The isogenic control and Wolfram iPSCs Wolf-2010-11.1, Wolf-2011-13 and Wolf-2010-9.4 were differentiated into islet-like aggregates [35–38]. At the end of differentiation, control and WFS1-deficient iPSC-derived beta cell aggregates contained comparable proportions of beta (36%), alpha (14%), delta (0.4%) and polyhormonal cells (9%) (Fig. 5a,b). The remaining 40% of the cells were negative for insulin, glucagon or somatostatin and are probably stem-cell-derived enterochromaffin-like cells [43, 44]. mRNA expression of key differentiation markers was similar between control and Wolfram syndrome lines and comparable to primary human islets (ESM Fig. 5).

Time course studies in iPSC whole aggregates showed that exenatide protected WFS1-deficient cells from tunicamycin-induced apoptosis (Fig. 5c–e). Dispersed Wolfram iPSC-derived beta cells showed higher tunicamycin-induced apoptosis compared with control cells (Fig. 5f), in keeping with our observations in EndoC-βH1 and primary human islet cells. In dispersed WFS1-deficient iPSC-derived beta cells, basal and tunicamycin-induced apoptosis was reduced by both exenatide and forskolin (Fig. 5f). In keeping with the findings in EndoC-βH1 cells, the drugs did not reduce ER stress markers (ESM Fig. 6a–c) at the time point analysed.

We next examined by Seahorse whether exenatide or forskolin improved mitochondrial function. The 72 h treatment did not modify mitochondrial functionality in Wolfram or control iPSC-derived beta cells (ESM Fig. 6d,e) and, contrary to earlier findings [34], Wolfram and control iPSC-derived aggregates had comparable OCR profiles (ESM Fig. 6d,e) and ER stress levels (ESM Fig. 6a–c). In EndoC-βH1 cells, *WFS1* silencing did not impair mitochondrial function either, and a 72 h exenatide or forskolin treatment also did not affect it (ESM Fig. 6f,g). Altogether, these data suggest that, different from neurons, a profound loss in WFS1 expression and intense ER stress is probably needed in beta cells to induce mitochondrial failure. Considering that the Wolfram syndrome iPSCs used here had different *WFS1* mutations from the ones utilised in the previous study [34], the ER stress-mediated mitochondrial failure in iPSC-derived beta cells may also depend on specific *WFS1* pathogenic variants.

### Dulaglutide improves Wolfram syndrome iPSC-derived beta cell function

**Fig. 6:**
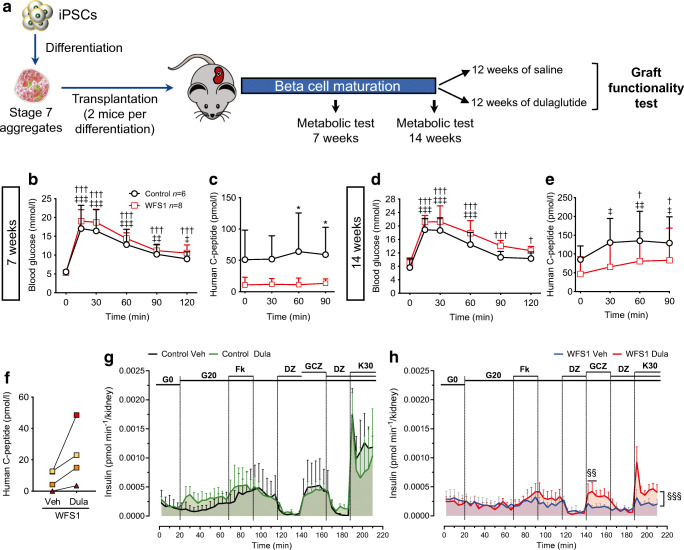
Dulaglutide improves Wolfram syndrome iPSC beta-like cell function. (**a**) Schematic representation of the experiment. iPSC-derived stage 7 control (Wolf-9.4-Corr-2G6.1, *n*=3) or Wolfram syndrome aggregates (Wolf-2010-9.4, *n*=3 and Wolf-2010-11.1, *n*=1 differentiation) were transplanted under the kidney capsule of *Rag2* KO mice (2 mice per differentiation). (**b**–**e**) IPGTT glucose levels and human C-peptide secretion evaluated 7 and 14 weeks after transplantation (*n*=6 mice transplanted with control aggregates, and *n*=8 mice transplanted with WFS1 aggregates). (**f**–**h**) Fourteen weeks after transplantation one mouse from each pair was randomised to either the dulaglutide (1 mg/kg every 4 days) or saline (Veh) group for 12 weeks. Results are expressed as mean and SD. (**f**) Non-fasting human C-peptide levels in WFS1-transplanted mice after 12 weeks of dulaglutide or vehicle administration. Paired symbols represent iPSC beta cells from one differentiation. Wolf-2010-9.4 is represented with squares and Wolf-2010-11.1 with triangles. Insulin secretion by control (**g**) and WFS1-deficient (**h**) grafts during in situ kidney perifusion with medium containing 0 (G0) or 20 mmol/l glucose (G20) alone or combined with forskolin, diazoxide, gliclazide or KCl. WFS1, *n*=4 mice per group; control, *n*=2 per group. ^†^*p*<0.05, ^†††^*p*<0.001 other time points vs T0 in WFS1; ^‡^*p*<0.05, ^‡‡^*p*<0.01, ^‡‡‡^*p*<0.001 other time points vs T0 in control; *p<0.05 control vs WFS1; ^§§^*p*<0.01, ^§§§^*p*<0.001 WFS1 Veh vs WFS1 Dula by two-way ANOVA with Sidak’s or Dunnet’s correction for multiple comparisons. Dula, dulaglutide; DZ, diazoxide; GCZ, glicazide; K30, potassium chloride 30 mmol/l; T0, time zero. Panel (**a**) was generated using Servier Medical Art, provided by Servier, licensed under a Creative Commons Attribution 3.0 Unported License (https://creativecommons.org/licenses/by/3.0/)

To extend our findings to an in vivo humanised Wolfram syndrome model, we transplanted iPSC-derived beta cell aggregates from two WFS1-deficient individuals and an isogenic control under the kidney capsule of immunodeficient *Rag2* KO mice (Fig. 6a). Human C-peptide secretion from control or Wolfram beta cells was stimulated by glucose 14 weeks but not 7 weeks after transplantation (Fig. 6b–e). The proportion of beta cells was similar in control and Wolfram iPSC-derived aggregates (32±0.2% vs 34±0.8%, respectively), but the Wolfram-derived cells secreted less C-peptide, consistent with beta cell dysfunction (Fig. 6c,e).

Fourteen weeks after transplantation, mice transplanted with aggregates from the same differentiation were randomised to 12 week dulaglutide or saline injection. In mice transplanted with Wolfram iPSC-derived beta cells, 12 week dulaglutide treatment enhanced non-fasting human C-peptide levels (Fig. 6f). To document functional changes with more granularity, we performed in situ graft perifusion studies. Since the iPSC-derived beta cells have limited glucose responsiveness, the insulin secretion in response to glucose was assessed between 0 and 20 mmol/l glucose. In saline-treated mice, Wolfram iPSC-derived beta cells failed to secrete insulin in response to any of the secretagogues tested (Fig. 6h). On the other hand, in dulaglutide-treated mice gliclazide and KCl, but not high glucose, stimulated insulin secretion, showing that GLP-1R agonists increase at least in part the function of beta cells from individuals affected by Wolfram syndrome (Fig. 6h). The lack of glucose responsiveness in this dynamic perifusion setting was also seen in control iPSC-derived beta cell grafts (Fig. 6g), suggesting that the grafted beta cells did not reach full maturity. Insulin and glucagon immunostaining of harvested grafts showed that dulaglutide did not modify beta cell but reduced alpha cell proportions in WFS1-deficient grafts (ESM Fig. 7).

### Exenatide protects Wolfram syndrome iPSC-derived NPCs and cerebellar neurons

**Fig. 7:**
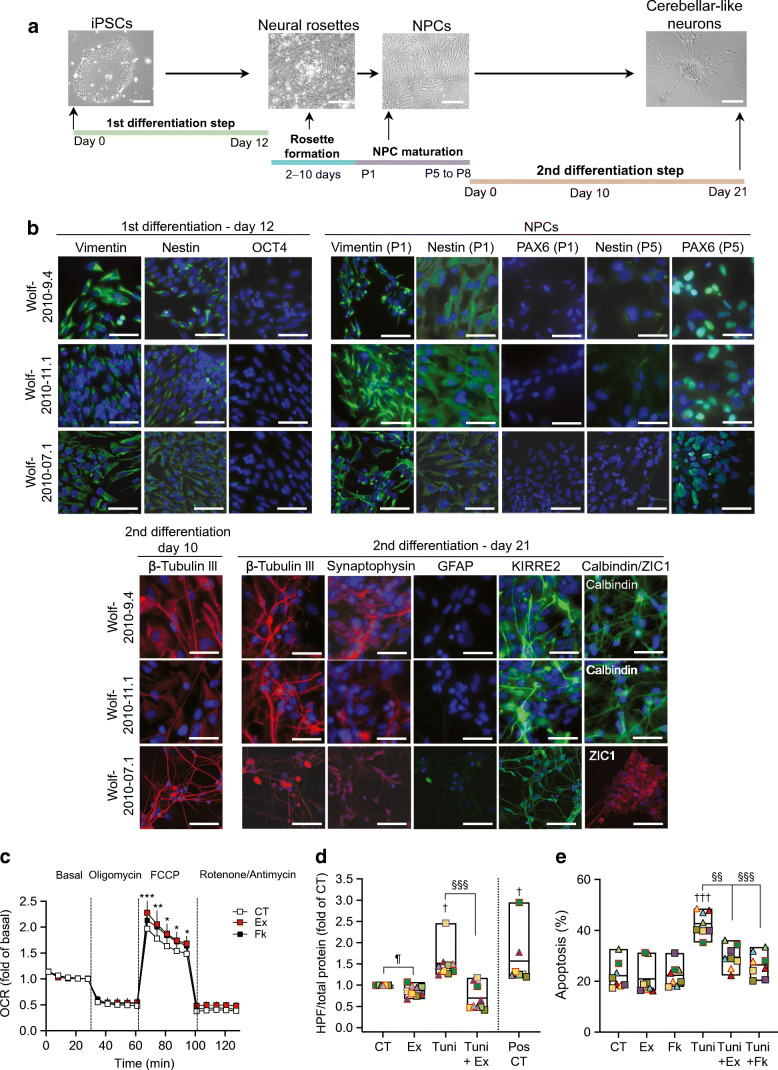
Exenatide protects Wolfram syndrome iPSC-derived neural precursors and cerebellar neurons. iPSCs Wolf 2010-9.4, Wolf 2010-11.1 and Wolf-2010-07.1 were differentiated into cerebellar neuron-like cells. (**a**) Differentiation timeline with representative phase contrast images of iPSCs (scale bar, 120 μm), neural rosettes, NPCs and cerebellar neuron-like cells (scale bars, 50 μm). (**b**) Representative immunofluorescence images for key markers OCT4, nestin, vimentin, PAX6, β-tubulin III, synaptophysin, GFAP, KIRRE2, ZIC1 and calbindin at day 12 of the first differentiation, in early NPCs (passage 1, P1) and at days 10 and 21 of the second differentiation. Scale bars, 30 μm. (**c**) Mitochondrial function by Seahorse in NPCs (*n*=3) and cerebellar neuron-like cells (*n*=3) exposed or not for 72 h to exenatide (500 nmol/l) or forskolin (20 μmol/l). Mitochondrial respiration was measured basally and after sequential injection of 20 mmol/l glucose, ATP synthase inhibitor oligomycin (5 μmol/l), oxidative phosphorylation uncoupler FCCP (4 μmol/l), and electron transport chain inhibitors rotenone and antimycin (1 μmol/l). Results are shown as means ± SEM of the two cell types together. (**d**, **e**) WFS1 iPSC-derived NPCs (squares) and cerebellar neuron-like cells (triangles) were exposed or not (CT) for 48 h to tunicamycin, alone or together with exenatide or forskolin. (**d**) Oxidative stress was measured by HPF. Menadione-treated cells were used as a positive control. (**e**) Apoptosis was determined by Hoechst 33342/propidium iodide. Extremities of floating bars are maximal and minimal values; horizontal line shows median. Symbols with the same colour come from one differentiation. *p<0.05, **p<0.01, ***p<0.001 Ex vs CT, ^†^*p*<0.05, ^†††^*p*<0.001 treated vs CT; ^§§^*p*<0.01, ^§§§^*p*<0.001 Tuni vs Tuni+Ex or Tuni+Fk; ^¶^*p*<0.05 Ex vs CT, by two-way or one-way ANOVA followed by Dunnet or Holm–Sidak correction for multiple comparisons. CT, control (vehicle); Ex, exenatide; Fk, forskolin; GFAP, glial fibrillary acidic protein; P, passage; PAX6, paired box protein 6; Pos, positive control; Tuni, tunicamycin

To assess whether these protective effects extend beyond beta cells, we generated iPSC-derived NPCs and cerebellar neurons using the iPSC lines from individuals with Wolfram syndrome (Fig. 7a). Because the isogenic control iPSCs failed to differentiate into cerebellar neurons (data not shown), and we did not have age-matched or family control iPSCs, the experiments were done using Wolfram cells only. The first step of the differentiation generated neural stem cells, positive for vimentin and nestin and negative for octamer binding protein 4 (OCT4) (Fig. 7b). Next, these cells were differentiated into neural stem cells which form neural rosettes and further mature into NPCs and cerebellar neuron-like cells. These immature cerebellar neurons were positive for cerebellar-neuroepithelial marker Kin of IRRE-like protein 2 (KIRRE2), granular cell marker zinc finger protein ZIC 1 (ZIC1), Purkinje cell marker calbindin, neuronal marker β-tubulin III and pre-synaptic marker synaptophysin, demonstrating cerebellar identity (Fig. 7b).

Exenatide and forskolin improved the maximal respiratory capacity of Wolfram cells, evidenced by enhanced OCR in response to mitochondrial uncoupler FCCP (Fig. 7c). Exenatide significantly reduced basal and tunicamycin-induced reactive oxygen species production in iPSC-derived NPCs and cerebellar neurons (Fig. 7d). Moreover, exenatide and forskolin protected these cells from tunicamycin-induced apoptosis. Exenatide hence improves mitochondrial function, reduces oxidative stress and protects WFS1-deficient iPSC-derived neurons from apoptosis, supporting the therapeutic potential of GLP-1R agonists to prevent cerebellar neurodegeneration in Wolfram syndrome.

## Discussion

Wolfram syndrome is a life-threatening disorder that has no preventive, therapeutic or curative options. Several drug repurposing strategies are currently under investigation [45–47]. Exenatide and liraglutide were shown to prevent and reverse diabetes, enhance insulin secretion, and reduce neuroinflammation and vision loss in rodent models of Wolfram syndrome [21–26]. Here, we confirmed that the long-acting GLP-1R agonist dulaglutide also prevents and reverses glucose intolerance/diabetes in *Wfs1* KO mice by enhancing beta cell function. More importantly, we demonstrate that short- and long-acting GLP-1R agonists confer protection in different human models of Wolfram syndrome, both in vitro in clonal and primary human beta cells, Wolfram iPSC-derived beta cells, NPCs and cerebellar neurons, and in vivo in humanised mice grafted with Wolfram iPSC-derived beta cells.

In vitro, exenatide and the cAMP inducer forskolin prevented ER stress-induced beta cell apoptosis without alleviating ER stress. This antiapoptotic effect is probably the consequence of the JunB/ATF3 pathway activation shown to be protective for pancreatic beta cells [42]. Exenatide and forskolin further improve mitochondrial function, reduce oxidative stress and prevent apoptosis in Wolfram iPSC-derived NPCs and cerebellar neurons, indicating that GLP-1R agonists have indeed therapeutic potential to prevent or delay neurodegeneration in Wolfram syndrome. GLP-1R agonists also showed beneficial effects in other neurodegenerative diseases such as Friedreich’s ataxia, Parkinson’s and Alzheimer’s disease, where they reduce neuronal degeneration and loss, alleviate oxidative stress, attenuate behavioural deficits, decrease neuroinflammation or reduce pathological protein aggregation, among others [19, 36].

In vivo*,* a clear improvement in beta cell function was evident in *Wfs1* KO mice at different ages. We did not find evidence of antiapoptotic effects, as 8 week dulaglutide administration did not prevent beta cell loss. Importantly, in the humanised mouse model of Wolfram syndrome, dulaglutide improved the function of iPSC-derived beta cells. Recent studies showed that liraglutide also provides extra-pancreatic protection in *Wfs1* KO rats, reducing neuroinflammation, improving learning capacity, preventing optic nerve degeneration and reducing hippocampal lateral ventricle size [24, 26].

For GLP-1R agonists to exert pleiotropic effects in Wolfram syndrome, the drugs should reach all affected tissues, including the brain. Dulaglutide is large (>59 kDa), containing two GLP-1 molecules fused to the IgG4 Fc domain [16], which might hinder blood–brain barrier crossing [16, 20]. Facilitating blood–brain barrier crossing of GLP-1R agonists is sought after for other neurodegenerative diseases [36, 48]. Solving this problem may also be helpful in Wolfram’s syndrome.

Sporadic clinical data on GLP-1R agonist use in people with Wolfram syndrome are accumulating. A 16 week liraglutide administration in an adult with Wolfram syndrome transiently (4 weeks) improved glucose tolerance [21], while in another individual with an autosomal dominant WFS1 mutation insulin therapy could be discontinued with GLP-1 analogue treatment [49]. A 7–27 week liraglutide administration in four children with Wolfram syndrome [50] led to increased C-peptide secretion in the first 7 weeks of treatment but in two individuals studied for a longer time C-peptide levels declined thereafter [50]. A stabilisation of neuro-ophthalmological disease was reported with liraglutide. Despite these promising data, controlled trials are needed to solidly assess safety and efficacy for glucose control. These could be designed as *n*=1 trials, using a crossover format that is well suited to rare diseases. Conclusive data on the impact of GLP-1R agonists on neurodegenerative features of Wolfram syndrome may require long-term, double-blind, randomised, placebo-controlled trials or data extraction from the electronic medical records of individuals with Wolfram syndrome who have received or not received GLP-1R agonists.

In conclusion, we provide evidence for the beneficial impact of both short- and long-acting GLP-1R agonists in human pancreatic beta cells and neurons and humanised mice, thereby providing a strong preclinical basis to clinically test these drugs in people with Wolfram syndrome.

## Supplementary Information

ESM (PDF 4470 kb)

## Data Availability

Data sharing is not applicable to this article as no datasets were generated or analysed during the current study.

## Abbreviations

ATF3: Activating transcription factor 3
BiP: Endoplasmic reticulum chaperone BiP
ER: Endoplasmic reticulum
GLP-1: Glucagon-like peptide 1
GLP-1R: Glucagon-like peptide 1 receptor
HPF: Hydroxyphenyl fluorescein
iPSC: Induced pluripotent stem cell
JunB: Transcription factor JunB
KIRRE2: Kin of IRRE-like protein 2
KO: Knockout
NPC: Neural precursor cell
OCR: Oxygen consumption rate
OCT4: Octamer binding protein 4
siCT: Control siRNA
WFS1: Wolframin
WT: Wild-type
ZIC1: Zinc finger protein ZIC 1
