# Supplementary material for: GLP-1R agonists demonstrate potential to treat Wolfram syndrome in human preclinical models
Source: Diabetologia. 2023 Mar 30;66(7):1306–21. doi: 10.1007/s00125-023-05905-8 (PMC10244297; doi:10.1007/s00125-023-05905-8)
Supplement: Supplementary file 1 — (PDF 4470 kb) [file 125_2023_5905_MOESM1_ESM.pdf]

ESM

Methods

Ethical approvals

All mouse experiments were approved by the Commission d'Ethique et du Bien Être Animal CEBEA, Faculty of Medicine, Université Libre de Bruxelles. CEBEA follows the European Convention for the Protection of Vertebrate Animals used for Experimental and other Scientific Purposes (European Treaty Series No.123).

Human pancreas not suitable for clinical use were collected from nondiabetic brain-dead organ donors in Pisa, Italy [1], Edmonton, Canada (http://www.bcell.org/adi-isletcore.html), or Barcelona, Spain with approval of the Human Research Ethics Committees of the University of Pisa, Alberta (Pro00013094), and Hospital Universitari Bellvitge (Barcelona) (PR239/13). Next-of-kin provided signed informed consent for the use of pancreas for research.

Skin biopsies were obtained from Wolfram syndrome individuals after signed informed consent by the patient or a legally responsible relative, with approval by the Columbia Institutional Review Board and Embryonic Stem Cell Research Oversight Committee. Fibroblasts were reprogrammed into induced pluripotent stem cells (iPSCs), and one iPSC line was corrected by CRISPR-Cas9 to generate an isogenic control [2]. iPSCs were differentiated into beta cells and neurons, with approval of the Ethical Committee of Erasmus Hospital, Université Libre de Bruxelles (P2019/498).

Mice

Genotyping was performed as described [3]. Immunodeficient B6(Cg)-Rag2$^{tm1.1Cgn}$/J (*Rag2* KO) mice on C57BL/6J background from the Jackson Laboratories (N° 008449) were used for human iPSC-derived beta cell transplantation. Mice were housed in the certified animal facility, Faculty of Medicine, Université Libre de Bruxelles, with 12 h light/dark cycle and ad libitum access to regular chow (Safe Diets A03).

GLP-1R agonist administration and *in vivo* metabolic studies

Male and female *Wfs1* KO and WT littermate mice received dulaglutide (Trulicity, Lilly, USA, 1 mg/kg every 4 days) or vehicle (saline solution) by intraperitoneal (IP) injection. Inclusion criteria: only animals with confirmed genotype (Wfs1 KO or WT) were used for the study. No exclusion criteria were established. The treatment was initiated at 4, 7 or 21 weeks of age. Because not all mice were available at once, we used a multi-batch study design. Twelve batches of 3 to 8 mice with variable combinations of male and female WT and *Wfs1* KO were allocated to dulaglutide or vehicle treatment by simple randomization (lottery). Due to the small size of the batches the basal metabolic data could not be taken into consideration to allocate the mice into the treatment groups. Treatment order and parameter assessment was random. A single animal was considered as the experimental unit. A priori sample size was calculated using G*power 3.1.9.6 (https://www.psychologie.hhu.de/arbeitsgruppen/allgemeine-psychologie-und-arbeitspsychologie/gpower) considering a standardized effect size of 1.99 reflecting the change in glycemia (considered as the primary outcome) induced by 4-week dulaglutide administration in *Wfs1* KO mice. A minimum of 6 mice per group were needed to detect

this difference with $p<0.05$ and a power of 85%. 48 h after dulaglutide or saline injection, IPGTT and ITT [4] were done by a researcher blinded for treatment. Data analysis was not blinded. For the IPGTT, glucose (2 mg/g body weight) was injected IP after 6 h fasting. Tail vein glycaemia was measured by Accu-Chek Aviva Nano (Roche, Switzerland) at times 0, 15, 30, 60, 90 and 120 minutes, and plasma insulin was measured by ultrasensitive mouse insulin ELISA (Crystal Chem, USA). Glucose tolerance was calculated as area under the curve (AUC) of IPGTT glycemia from 0 to 120 minutes. For the ITT, human recombinant insulin (Actrapid, Novo Nordisk, Danemark) was injected IP in 4 h-fasted mice. Due to gender differences in insulin sensitivity Actrapid was used at 0.75 mU/g body weight in males, and 0.4 mU/g in females. Tail vein glycaemia was measured at times 0, 15, 30, 60, 90 and 120 minutes. Insulin sensitivity was calculated as area over the curve (AOC) of ITT glycaemia from 0 to 30 minutes. The insulinogenic index was calculated as incremental insulin (ΔI) over incremental glucose (ΔG) in the first 15 minutes of IPGTT. Beta cell function was calculated as insulinogenic index × insulin sensitivity (ITT AOC) [4].

EndoC-βH1, human islet cells and iPSC lines

The EndoC-βH1 cells were free of mycoplasma contamination as determined by the MycoAlert Mycoplasma Detection Kit (Lonza, Switzerland).

Human islets from nondiabetic organ donors (*n*= 5, age 58 ± 9 years, body mass index 24 ± 1 kg/m^2, cause of death: cerebral hemorrhage and stroke) were isolated in Pisa, Italy, Edmonton, Canada, or Barcelona, Spain, by collagenase digestion and density gradient purification as described [5, 6]. Islets were shipped to Brussels and cultured in

Ham's F10 (Gibco, USA) containing 6.1 mmol/l glucose (Sigma-Aldrich, USA), 10% FBS (Gibco), 2 mmol/l GlutaMAX (Gibco), 50 µmol/l 3-isobutyl-1-methylxanthine (Sigma-Aldrich), 1% BSA (Roche), 50 U/ml penicillin and 50 µg/ml streptomycin (Lonza), and dispersed as previously described [7]. Beta cell purity, determined by insulin immunofluorescence, was 40±4%. EndoC-βH1 and dispersed islet cells were transfected overnight with 30 nmol/l control small interfering RNA (siRNA) (siCT, Qiagen, USA) that does not interfere with cell function or gene expression or with two siRNAs targeting human *WFS1* (Invitrogen, USA). siRNA sequences and lipofectamine concentrations are described in ESM Tables 1 and 2.

iPSC lines (Wolf-2010-07.1, Wolf-2010-11.1, Wolf-2011-13.2, and Wolf-2010-9.4) from four Wolfram syndrome patients and one isogenic control line (Wolf-9.4-Corr-2G6.1), generated by CRISPR-Cas9 editing of Wolf-2010-9.4, were cultured in Matrigel-coated plates (Corning BV, Life Sciences, USA) in E8 medium (Life Technologies, USA) without antibiotics and passaged with 0.5 mmol/l EDTA (Life Technologies) twice weekly [8]. *WFS1* variants and patient characteristics are shown in ESM Table 3.

iPSCs were differentiated into beta-like cell aggregates using pour previously validated 7-stage protocol. Differentiation efficiency was assessed by analyzing key differentiation markers by real-time PCR and immunofluorescence [4, 8, 9], using primers listed in ESM Tables 4 and 5, and antibodies listed in ESM Table 6.

For iPSCs differentiation into cerebellar neuron-like cells, iPSCs were seeded in Matrigel-coated 6-well plates at 30-40% confluency and cultured in a 1:1 mixture of DMEM/F12

and neurobasal media (Gibco, USA) supplemented with additives along the 12-day differentiation (ESM Table 7). At day 12, cells were split using Accutase (Capricorn Scientific, Germany) and plated at low density ($3x10^6$ cells) in Matrigel-coated 10-cm plates in neural precursor cell (NPC) medium (ESM Table 8) until the appearance of neural stem cells (NSCs) forming neural rosettes (day 2 to 10). Rosettes were manually picked, transferred to new Matrigel-coated plates. The NSCs were then matured into NPCs and amplified in the same medium. NPCs expressed neural precursor markers PAX6, vimentin and nestin and were negative for the pluripotency marker OCT4. After 5 passages, the cells were either frozen, plated for experiments at cell densities described in ESM Table 9, or plated for cerebellar neuron differentiation. For the latter, NPCs were split using Accutase and plated at 80% confluency in NPC medium (overnight) in Matrigel-coated 6-well plates. Then, cells were grown for 10 days in second step differentiation medium (ESM Table 10), changed every second day. On day 11 cells were split using Accutase, plated in Matrigel-coated plates (cell densities described in ESM Table 11) and cultured for an additional 10 days with medium changes every second day. The resulting immature cerebellar neurons were positive for cerebellar markers KIRRE2, ZIC 1 and calbindin, neuronal marker β-tubulin III, and postsynaptic marker synaptophysin [10, 11] and negative for astrocyte marker GFAP.

Cell treatments and cell death assays

EndoC-βH1 cells, dispersed human islets, iPSC-derived beta cells, NPCs and cerebellar neurons were exposed to the ER stressors tunicamycin (5 μg/ml), thapsigargin (1 μmol/l) or brefeldin A (0.05 μg/ml), alone or combined with exenatide (50 nmol/l in beta cells, 500

nmol/l in neurons) or forskolin (20 µmol/l, all from Sigma-Aldrich). EndoC-βH1 cells were pretreated with exenatide or forskolin for 2 h, other cell types for 24 h. EndoC-βH1 medium contained 2% FBS [12], human islet and iPSC-beta cell medium was Ham's F10 containing 6.1 mmol/l glucose, 0.75% FFA-free BSA (Roche), 2 mM glutaMAX, 50 μg/ml streptomycin, 50 U/ml penicillin. Tunicamycin, exenatide and forskolin treatments in iPSC-derived NPCs and cerebellar neurons were performed in DMEM/F12 or neurobasal media, respectively, supplemented with 1X MEM non-essential aminoacids (Gibco), glutaMAX, and 50 μg/ml streptomycin, 50 U/ml penicillin.

Cell death was assessed by fluorescence microscopy counting after staining with DNA binding dyes Hoechst 33342 (5 μg/ml, Sigma-Aldrich) and propidium iodide (5 μg/ml, Sigma-Aldrich). At least 600 cells were counted by two observers, one of them unaware of experimental conditions. Early and late apoptosis was also assessed by Real time-Glo AnnexinV apoptosis and necrosis assay (Promega, USA). The data is expressed as Fold of Basal (T0) apoptosis in each condition.

iPSC-beta cell transplantation into immunodeficient mice

iPSC-derived beta cellaggregates were transplanted into 5–7-week-old male *Rag2* KO mice. Mice were anesthetized by IP injection of ketamine (100 mg/kg, Nimatek, Dechra, UK) and xylazine (5 mg/kg, Rompun, Bayer, Germany), and aggregates were transplanted under the kidney capsule using a 10 µl precision pipet. Paracetamol (100 mg/l drinking water) was given as analgesic one day prior to and during the 10 days following the surgery. Animals were monitored daily. 1000 aggregates from one

differentiation were transplanted into two mice. IPGTT was performed 7 and 14 weeks after transplantation, and plasma C-peptide measured by ultrasensitive human C-peptide ELISA (Mercodia, Sweden). Fourteen weeks after transplantation, mice were allocated to IP dulaglutide (1 mg/kg every 4 days) or vehicle injection for 12 weeks by simple randomization.

To assess grafted iPSC-beta cell function, the kidney was perfused (1 ml/min flow rate) *in situ* at 37°C in a single-pass circuit through the renal artery. The abdominal aorta was ligated above the coeliac trunk, a catheter inserted, and the venous effluent collected by another catheter inserted in the renal vein as previously described [13]. To avoid coagulation, the kidney was first perfused with 1 ml heparinized (50 U/ml) PBS. After 20 minutes equilibration in basal perifusion solution, the kidney was sequentially perfused with solution containing 0 (G0) or 20 mmol/l glucose (G20) alone or combined with forskolin (1 µmol/l), gliclazide (25 µmol/l), diazoxide (250 µmol/l) or KCl (30 mmol/l). Samples were collected every 4 minutes and human insulin was measured by radioimmunoassay [14].

Immunofluorescence, Western blott, real-time PCR and ROS detection

For immunostaining cells were fixed in 4% formaldehyde for 15–20 minutes, permeabilized with 0.5% Triton X-100 for 10 minutes, blocked with UltraV block (Thermo Fisher Scientific, USA) for 10 minutes, and incubated overnight at 4°C with primary antibodies diluted in PBS with 0.1% Tween. Samples were washed and incubated for 30 minutes at room temperature with fluorescent secondary antibodies, mounted in

Vectashield Vibrance Antifade Mounting medium with Dapi (Vector Laboratories, USA) and covered with glass coverslips.

Formalin-fixed paraffin-embedded mouse pancreas and iPSC-beta cell grafts were cut using microtome into 5-μm-thick sections. Sections were de-paraffinized with xylene and re-hydrated through descending graded alcohols to water. Antigens were retrieved by immersing slides in 10 mmol/l sodium citrate buffer pH 6 and microwaving until boiling. Samples were blocked and processed as described above. Antibodies and dilutions are provided in ESM Table 5. Pancreatic area was measured using ImageJ software (https://imagej.nih.gov/ij/index.html).

Total protein was extracted from EndoC-βH1 cells in RIPA buffer (Sigma-Aldrich) containing protease inhibitors (cOmplete™, EDTA-free protease inhibitors, Roche) or directly collected in Laemnly buffer 1x. In RIPA Buffer samples, protein was quantified by BCA method (Thermo Fisher Scientific). 20 µg protein was loaded on 10% SDS-polyacrylamide gels or in precast 7–15% gradient SDS-polyacrylamide gels (Bio-Rad). Proteins were transferred to nitrocellulose membranes, blocked with 5% nonfat milk, and incubated overnight with primary antibodies diluted in the same solution. The membranes were washed with PBS 0.1% Tween-20 and incubated with horseradish peroxidase–conjugated secondary antibodies (ESM Table 6). After washing, proteins were detected using SuperSignal West Femto chemiluminescent substrate (Thermo Fisher Scientific) in ChemiDoc XRS+ system (Bio-Rad) and quantified using Image Lab software.

mRNA was isolated using Dynabeads mRNA DIRECT (Thermo Fischer Scientific) and reverse transcribed. Gene expression was assessed by real-time PCR using Q SYBR

Green Supermix on a MyiQ2 single-color real-time PCR system (Bio-Rad). Gene expression was calculated as copies/µl using a standard curve (prepared in conventional PCR). *GAPDH* and/or beta actin *(ACTB)* were used as reference genes. Primer sequences are provided in ESM Table 4 and 5.

Cells seeded in black-bottom plates were incubated with the oxidation-sensitive fluorescent probe HPF (10 µmol/l, Invitrogen, USA) for 30 minutes and washed twice with PBS. Fluorescence was measured in VICTOR multilabel plate reader (Perkin-Elmer, USA) using excitation and emission spectra of 485 and 535 nm. Menadione (15 µmol/l for 2 h) was used as positive control.

Mitochondrial respiration

Mitochondrial function was assessed by measuring oxygen consumption rate (OCR) in a Seahorse XFp Extracellular Flux Analyzer (Agilent, USA). Dispersed iPSC-derived beta cells were exposed or not to exenatide or forskolin for 72 h and then preincubated for 1 h at 37°C in a non-CO_2 incubator in KREBS buffer (20 mmol/l Hepes, 2.5 mmol/l $CaCl_2$, 1.16 mmol/l $MgS0_4$, 1.2 mmol/l KH_2PO_4, 4.7 mmol/l KCl and 114 mmol/l NaCl) at pH 7.4 and with 0.2% FFA-free BSA. Mitochondrial respiration was measured basally and after sequential injection of 20 mM glucose, ATP synthase inhibitor oligomycin (5 µmol/l), oxidative phosphorylation uncoupler carbonyl cyanide-p-trifluoromethoxyphenylhydrazone (FCCP, 4 µmol/l), and electron transport chain inhibitors rotenone and antimycin (1 µmol/l). NPCs and cerebellar neurons were similarly exposed to exenatide/forskolin and preincubated in Agilent Seahorse XF DMEM medium pH 7.4 supplemented with 10 mmol/l glucose and 2 mmol/l glutaMAX. Mitochondrial

respiration was measured basally and after injection of 2 µmol/l oligomycin, 2 µmol/l FCCP, and 1 µmol/l rotenone plus antimycin A. OCR data were normalized to the last basal reading in each sample.

ESM Tables

ESM Table 1. Human WFS1 siRNA sequences

siRNA	Sequence
siWFS1#1	CAGCAGCGAGUCCAAGAACUA
siWFS1#2	CCGCGUGACUGACAUCGACAA

ESM Table 2. Transfection conditions for EndoC-βH1 cells and human islets

Cell type	Transfected sequence	Lipofectamine transfection reagent	Transfection reagent volume per 100 µl
Human islets	siRNA	RNAiMAX (Invitrogen, #13778150)	0.4
EndoC-βH1	siRNA	RNAiMAX (Invitrogen, #13778150)	0.2

ESM Table 3. iPSC line characteristics

iPSC line	Source	Genotype	mutation in allele 1	mutation in allele 2	Sex	Age at biopsy	Age at onset of DM	Age at onset of OA	Age at Deaf	DI
Wolf-2010-07.1	Fumihiko Urano	WFS1	c.2002C>T p.Q668X	c.2002C>T p.Q668X	M	9	2,7	NA	7	7
Wolf-2010-11.1	Fumihiko Urano	WFS1	c.376G>A p.A126T	c.1838G>A p.W613X	M	10	7,5	6	8	10
Wolf-2011-13.2	Fumihiko Urano	WFS1	c.599delT p.L200fs286Stop	c.2254G>T pE752Stop	F	7	4,8	5,2	6	7,5
Wolf-2010-9.4	Fumihiko Urano	WFS1	c.376G> p.A126T	c.1838G>A p.W613X	M	16	10,8	11	N	4

Wolf-9.4-Corr2G6.1	Fumihiko Urano	Isogenic control of Wolf-2010-9.4	c.376G>A p.A126T	c.1838G>A p.W613X	M					

DM, diabetes mellitus; OA, optic atrophy; Deaf, deafness; DI, diabetes insipidus.

ESM Table 4. List of real time PCR primers

Species	Gene	Forward sequence (5'→3')	Reverse sequence (5'→3')
Human	*WFS1*	GCTTGGCGGACAGAAGAG	ATGACCAGGGCTGCCTTG
Human	*GAPDH*	CAGCCTCAAGATCATCAGCA	TGTGGTCATGAGTCCTTCCA
Human	*ACTB*	CTGTACGCCAACACAGTGCT	GCTCAGGAGGAGCAATGATC
Human	*PDX1*	AAAGCTCACGCGTGGAAA	GCCGTGAGATGTACTTGTTGA
Human	*NKX6.1*	GGGCTCGTTTGGCCTATT	CGTGCTTCTTCCTCCACTT
Human	*NKX2.2*	GAACCCCTTCTACGACAGCA	ACCGTGCAGGGAGTACTGAA
Human	*NEUROD1*	CTATCACTGCTCAGGACCTACT	CCACTCTCGCTGTACGATTT
Human	*INS*	CCAGCCGCAGCCTTTGTGA	CCAGCTCCACCTGCCCCA
Human	*GGC*	GCTAAACAGAGCTGGAGAGTAT	AAGCCCTCTTTGGGAACTT
Human	*NGN3*	GACGACGCGAAGCTCACCAA	TACAAGCTGTGGTCCGCTAT
Human	*SOX9*	ATCAAGACGGAGCAGCTGAG	GGCTGTAGTGTGGGAGGTTG
Human	*GLP1R*	AAGGACAACTCCAGCCTGC	ATGATGTAGAGGAACAGGAG
Human	*CHOP*	Hs_DDIT3_1_SG Quantitect Primer Assay (Qiagen)	
Human	*BIP*	Hs_HSPA5_1_SG QuantiTect Primer Assay (Qiagen)	
Human	*ATF3*	GTAGCCCCTGAAGAAGATGAAAG	CTTCTCCGACTCTTTCTG
Human	*XBP1s*	CCGCAGCAGGTGCAGG	GAGTCAATACCGCCAGAATCCA
Human	*JUNB*	AAACTCCTGAAACCGAGCCT	GACCAGAAAAGTAGCTGCCG

ESM Table 5. List of primers to generate the real time PCR standards

Species	Gene	Forward sequence (5'→3')	Reverse sequence (5'→3')
Human	*WFS1*	AAGCACTACCTGCAGTTGGC	TGGCCGACATTCTCCAGCAG
Human	*GAPDH*	CTGAGAACGGGAAGCTTGTC	AGGTCAGGTCCACCACTGGAC
Human	*ACTB*	AAATCTGGCACCACACCTTC	CCGATCCACACGGAGTACTT
Human	*PDX1*	CTGCCTTTCCCATGGATGAA	CTTGATGTGTCTCTCGGTCAAG
Human	*NKX6.1*	AAACACACGAGACCCACTTT	GCTTATTGTAGTCGTCGTCCTC
Human	*NKX2.2*	AAGACGGGGTTTTCGGTCAA	TGTCATTGTCCGGTGACTCG
Human	*NEUROD1*	CTATCACTGCTCAGGACCTACT	GTCATCCTCCTCTTCCTCTTCT
Human	*INS*	TGTCCTTCTGCCATGGC	CCATCTCTCTCGGTGCA

Human	*GGC*	GGGAGAGGGAAGTCATTTGTAA	GTAGAACAGAGCAGGTGAAAAG
Human	*NGN3*	AAGAGCGAGTTGGCACTGAG	GAGCTGGAGTTCTGGTGGTC
Human	*SOX9*	TGGATGTCCAAGCAGG	GAGCTGGAGTTCTGGTGGTC
Human	*GLP1R*	AGAAATGGCGAGAATACCGAC	TGTGCTATACATCCACTTCAG
Human	*CHOP*	AGGCACTGAGCGTATCATGTT	CTGTTTCCGTTTCCTGGTTC
Human	*BIP*	TTCTTGTTGGTGGCTCGACT	GTCAGCATCTTGGTGGCTTT
Human	*ATF3*	TGTCAAGGAAGAGCTGAGGTTTG	CATCTTCTGGAGTCCTCCCATTC
Human	*XBP1s*	CCGCAGCAGGTGCAGG	GGGGCTTGGTATATATGTGG
Human	*JUNB*	TGGAACAGCCCTTCTACCAC	GGAGTAGCTGCTGAGGTTGG

ESM Table 6. Antibodies

Peptide/protein target	Antibody name	Manufacturer, catalogue #	Species raised in; mono- or polyclonal	Dilution	RRID
WFS1	Anti WFS1 polyclonal antibody	Invitrogen, #PA5-76065	Rabbit polyclonal	1:1000	AB_2719793
GAPDH	Anti-G3PDH Human Polyclonal Antibody	Trevigen, Gaithersburg, MD, USA, # 2275-PC-020	Rabbit polyclonal	1:1000	N/A
α-Tubulin	Monoclonal Anti-α-Tubulin antibody	Sigma-Aldrich, Bornem, Belgium, # T9026	Mouse monoclonal	1:5000	AB_477593
BiP	BiP (C50B12) Rabbit antibody	Cell signalling Danvers, MA UK, # 3177S	Rabbit polyclonal	1:1000	AB_2119845
ATF3	ATF-3 (D2Y5W) Rabbit antibody	Cell signalling Danvers, MA, UK, # 3359S)	Rabbit polyclonal	1:1000	AB_2799039
JUNB	JunB (C37F9) Rabbit antibody	Cell signalling Danvers, MA, UK, # 3753S	Rabbit polyclonal	1:1000	AB_2130002
OCT-3/4	Oct-4A (C30A3)Rabbit mAb	Cell signalling, #2840	Rabbit Monoclonal	1:400	N/A
SSEA-4	SSEA4 Monoclonal Antibody (MC-813-70)	Thermofisher Scientific, #MA1-021	Mouse Monoclonal	1:500	AB_2536687
TRA-1-60	TRA-1-60 Monoclonal Antibody (TRA-1-60)	Thermofisher Scientific, #MA1-023	Mouse Monoclonal	1:100	AB_2536699
Nanog	Nanog (D73G4)XP Rabbit mAb	Cell signalling, #4903	Rabbit,Monoclonal	1:400	AB_10559205
Insulin	Polyclonal Guinea Pig Anti- Insulin antibody	DAKO, Glostrup, Denmark, #A056401-2	Guinea pig polyclonal	Ready to use	AB_2617169
Glucagon	Anti-Glucagon-Antibody	Sigma-Aldrich, #SAB4501137	mouse monoclonal	1:100	AB_10761583
Human nucleoli	Recombinant Anti-Human Nucleoli antibody [NM95] - Nucleolar Marker	Abcam, #ab190710	Mouse monoclonal	1/200	N/A
Nestin	Anti-Nestin Antibody, clone 8B8, ZooMAb® Rabbit Monoclonal	Sigma-Aldrich, #ZRB69	Rabbit Monoclonal	1:200	N/A
Vimentin	Anti-Vimentin polyclonal antibody	Abcam, #137321	Rabbit polyclonal	1:250	N/A

β– tubulin III	Anti-β III Tubulin mAb	Promega, #G7121	Mouse monoclonal	1:200	AB_430874
PAX6	Purified anti-Pax-6 polyclonal antibody Clone 19013	Biolegend, #901301	Rabbit polyclonal	1:100	AB_2565003
Kirrel2	Kirrel2	Biosource, #MBS2523887	Rabbit polyclonal	1:100	N/A
ZIC 1	Anti ZIC1 polyclonal antibody	R &D systems, #AF4978	Goat polyclonal	1:150	N/A
GFAP	Anti GFAP antibody	Agilent, #Z0334	Rabbit polyclonal	1:1000	AB_10013382
Anti-mouse IgG	Peroxidase AffiniPure F(ab')2 Fragment Donkey Anti-Mouse IgG (H+L)	Jackson ImmunoResearch Laboratories, Wes Grove, PA, USA, #715-036-150	Rabbit polyclonal	1:5000	AB_2340773
Anti-rabbit IgG	Peroxidase AffiniPure F(ab')2 Fragment Donkey Anti-Rabbit IgG (H+L)	Jackson ImmunoResearch Laboratories, Wes Grove, PA, USA, #711-036-152	Rabbit polyclonal	1:5000	AB_2340590
Goat anti-guinea pig IgG	Donkey anti-Guinea Pig IgG, Secondary Antibody, Alexa Fluor 488	Jackson ImmunoResearch, #706-545-148	Donkey polyclonal	1:500	AB_2340472
Goat anti-rabbit IgG	Donkey Anti Rabbit (Alexa Fluor 488)	Jackson ImmunoResearch, #711-545-152	Donkey polyclonal	1:500	AB_2313584
Goat anti-rabbit IgG	Donkey Anti Rabbit (Rhodamine Red)	Jackson ImmunoResearch, #711-295-152	Donkey polyclonal	1:500	AB_2340613
Donkey Anti-Mouse IgG	Donkey anti-Mouse (Alexa Fluor 488)	Jackson ImmunoResearch, #715-545-151	Donkey polyclonal	1:500	AB_2341099
Donkey Anti-Mouse IgG	Donkey anti-Mouse (Rhodamine Red)	Jackson ImmunoResearch, #715-025-151	Donkey polyclonal	1:500	AB_2340767
Donkey Anti-Goat IgG	Donkey anti-Goat (Alexa Fluor 488)	Jackson ImmunoResearch, #711-545-147	Donkey polyclonal	1:500	AB_2336933
Donkey Anti-Guinea Pig IgG	Alexa Fluor488-conjugated AffiniPure Donkey Anti-Guinea Pig (H+L) antibody	Jackson ImmunoResearch Laboratories, Wes Grove, PA, USA, #706-545-148	Donkey polyclonal	1:500	AB_2340472

ESM Table 7. Media composition for the first 11 days of the cerebellar neuron differentiation

	**Media composition (final concentrations)**				
**Additives**	**For day 0 to 3 (Final conc)**	**For day 4 and 5 (Final conc)**	**For day 6 and 7 (Final conc)**	**For day 8 and 9 (Final conc)**	**For day 10 and 11 (Final conc)**
	DMEM/F-12 GlutaMAX supplement, Gibco #10565018 : Neurobasal Medium, Gibco # 21103049. (1:1)				
MEM-Non-essential aminoacids solution 100 X, Gibco # 11140050	1 X				
Insulin/Transferrin/Selenium (IST-G) 100 X, Gibco #4140004	1 X				
β-mercaptoethanol 50 mmol/l, Gibco #31350010	100 μmol/l				
Penicillin (5000 U/ml) Streptomycin (5000 μg/ml), Lonza #DE17-603E	5 U/ml Penicillin - 5 μg/ml Streptomycin				

SB431542, Stemcell,Canada, #72232 (20 mmol/l in DMSO)	10 µmol/l	7.5 µmol/l	5 µmol/l	2.5 µmol/l	/
LDN-193189, Selleckchem, USA #S2618 (1mmol/l in DMSO)	100 nmol/l	100 nmol/l	100 nmol/l	100 nmol/l	100 nmol/l
CHIR99021, Axon Medchem, USA #1386 (10 mmol/l in DMSO)	1.7 µmol/l	1.7 µmol/l	1.7 µmol/l	1.7 µmol/l	1.7 µmol/l
N2 supplement 100 X, Gibco #17502048	/	0.25 X	0.5 X	0.75 X	1 X
B27 supplement minus vitamin A 50 X, Gibco #12587010	/	0.25 X	0.5 X	0.75 X	1 X
Retinoic Acid, Sigma-Aldrich #R2625 (20 mmol/l in DMSO)	/	/	1 µmol/l	1 µmol/l	1 µmol/l
FGF-8B human recombinant, Peprotech, USA #AF-100-25 (100 µg/ml in PBS 0.1% BSA)	/	/	100 ng/ml	100 ng/ml	100 ng/ml
FGF-2 human recombinant, Peprotech #100-18B (20 µg/ml in PBS 0.1% BSA)	/	/	4 ng/ml	4 ng/ml	4 ng/ml

ESM Table 8. Media composition for neural precursor cell (NPC) culture

	**Media composition (final concentrations)**
Additives	DMEM/F-12 GlutaMAX supplement, Gibco #10565018
MEM-Non-essential aminoacids solution 100 X, Gibco # 11140050	1 X
Penicillin (5000 U/ml) Streptomycin (5000 µg/ml), Lonza #DE17-603E	5 U/ml Penicillin - 5 µg/ml Streptomycin
N2 supplement 100 X, Gibco #17502048	1 X
B27 supplement minus vitamin A 50 X, Gibco #12587010	1 X
EGF human recombinant, Stemcell #78006 (1mg/ml in water)	20 ng/ml
FGF-2 human recombinant, Peprotech #100-18B (20 µg/ml in PBS 0.1% BSA)	20 ng/ml

ESM Table 9: Number of NPCs plated

**Type of plate and application**	**Cell number per well**
96 well plate (apoptosis counting)	20,000 cells/well
96 well plate (qPCR)	45,000 cells/well
96 well black plate (oxidative stress measurement)	45,000 cells/well
Seahorse plate (mitochondrial function)	40,000 cells/well

8 well plate with square wells (Immunofluorescence)	50,000 cells/well

ESM Table 10. Media composition for the second step of the cerebellar neurons differentiation

	**Media composition (final concentrations)**			
**Additives**	**For day 1 and 2 (Final conc)**	**For day 3 to 6 (Final conc)**	**For day 7 to 14 (Final conc)**	**For day 15 to 21 (Final conc)**
	Neurobasal Medium (Gibco # 21103049)			
MEM-Non-essential aminoacids solution 100 X, Gibco # 11140050	1 X			
GlutaMAX supplement 100 X, Gibco #35050061	1 X			
Penicillin (5000 U/ml) Streptomycin (5000 µg/ml), Lonza #DE17-603E	5 U/ml Penicillin - 5 µg/ml Streptomycin			
N2 supplement 100 X, Gibco #17502048	1 X			
B27 supplement without vitamin A 50 X, Gibco #12587010	1 X			
FGF-8B human recombinant, Peprotech #AF-100-25 (100 µg/ml in PBS 0.1% BSA)	100 ng/ml	100 ng/ml	/	/
FGF-2 human recombinant, Peprotech #100-18B (20 µg/ml in PBS 0.1% BSA)	20 ng/ml	/	/	/
FGF-4 human recombinant, Peprotech #100-31 (100 µg/ml in PBS 0.1% BSA)	100 ng/ml	/	/	/
BDNF human recombinant, Peprotech #450-02 (100 µg/ml in PBS 0.1% BSA)	/	/	50 ng/ml	50 ng/ml
GDNF human recombinant, Peprotech #450-10 (20 µg/ml in PBS 0.1% BSA)	/	/	10 ng/ml	10 ng/ml
SAG, Sigma-Aldrich #SML1314 (100 µg/ml in PBS 0.1% BSA)	/	/	/	3 ng/ml
NT-3 human recombinant, Peprotech #450-03 (100 µg/ml in PBS 0.1% BSA)	/	/	/	100 ng/ml
KCL, Sigma-Aldrich #P5405 (2.5 mol/l in water)	/	/	/	25 mmol/l

ESM Table 11: Number of iPSC-derived cerebellar neurons plated

**Type of plate and application**	**Cell number per well**
96 well plate (apoptosis counting)	40,000 cells/well

96 well plate (qPCR)	90,000 cells/well
96 well black plate (oxidative stress measurement)	90,000 cells/well
Seahorse plate (mitochondrial function)	80,000 cells/well
8 well plate with square wells (immunofluorescence)	100,000 cells/well

References

[1] Marchetti P, Suleiman M, Marselli L (2018) Organ donor pancreases for the study of human islet cell histology and pathophysiology: a precious and valuable resource. Diabetologia 61(4): 770-774. 10.1007/s00125-018-4546-x

[2] Maxwell KG, Augsornworawat P, Velazco-Cruz L, et al. (2020) Gene-edited human stem cell-derived beta cells from a patient with monogenic diabetes reverse preexisting diabetes in mice. Sci Transl Med 12(540). 10.1126/scitranslmed.aax9106

[3] Koks S, Soomets U, Paya-Cano JL, et al. (2009) Wfs1 gene deletion causes growth retardation in mice and interferes with the growth hormone pathway. Physiol Genomics 37(3): 249-259. 10.1152/physiolgenomics.90407.2008

[4] Igoillo-Esteve M, Oliveira AF, Cosentino C, et al. (2019) Exenatide induces frataxin expression and improves mitochondrial function in Friedreich ataxia. JCI Insight. 10.1172/jci.insight.134221

[5] Marselli L, Suleiman M, Masini M, et al. (2014) Are we overestimating the loss of beta cells in type 2 diabetes? Diabetologia 57(2): 362-365. 10.1007/s00125-013-3098-3

[6] Shapiro AM, Lakey JR, Ryan EA, et al. (2000) Islet transplantation in seven patients with type 1 diabetes mellitus using a glucocorticoid-free immunosuppressive regimen. N Engl J Med 343(4): 230-238. 10.1056/NEJM200007273430401

[7] Santin I, Dos Santos RS, Eizirik DL (2016) Pancreatic Beta Cell Survival and Signaling Pathways: Effects of Type 1 Diabetes-Associated Genetic Variants. Methods Mol Biol 1433: 21-54. 10.1007/7651_2015_291

[8] Cosentino C, Toivonen S, Diaz Villamil E, et al. (2018) Pancreatic beta-cell tRNA hypomethylation and fragmentation link TRMT10A deficiency with diabetes. Nucleic Acids Res 46(19): 10302-10318. 10.1093/nar/gky839

[9] Demine S, Schiavo AA, Marin-Canas S, Marchetti P, Cnop M, Eizirik DL (2020) Pro-inflammatory cytokines induce cell death, inflammatory responses, and endoplasmic reticulum stress in human iPSC-derived beta cells. Stem Cell Res Ther 11(1): 7. 10.1186/s13287-019-1523-3

[10] Holmes DB, Heine VM (2017) Streamlined 3D Cerebellar Differentiation Protocol with Optional 2D Modification. J Vis Exp(130). 10.3791/56888

[11] Sundberg M, Tochitsky I, Buchholz DE, et al. (2018) Purkinje cells derived from TSC patients display hypoexcitability and synaptic deficits associated with reduced FMRP levels and reversed by rapamycin. Mol Psychiatry 23(11): 2167-2183. 10.1038/s41380-018-0018-4

[12] Brozzi F, Nardelli TR, Lopes M, et al. (2015) Cytokines induce endoplasmic reticulum stress in human, rat and mouse beta cells via different mechanisms. Diabetologia 58(10): 2307-2316. 10.1007/s00125-015-3669-6

[13] Jansson L, Eizirik DL, Pipeleers DG, Borg LA, Hellerstrom C, Andersson A (1995) Impairment of glucose-induced insulin secretion in human pancreatic islets transplanted to diabetic nude mice. J Clin Invest 96(2): 721-726. 10.1172/JCI118115

[14] Gilon P, Shepherd RM, Henquin JC (1993) Oscillations of secretion driven by oscillations of cytoplasmic Ca2+ as evidences in single pancreatic islets. J Biol Chem 268(30): 22265-22268

Checklist for reporting human islet preparations used in research

Adapted from Hart NJ, Powers AC (2018) Progress, challenges, and suggestions for using human islets to understand islet biology and human diabetes. Diabetologia https://doi.org/10.1007/s00125-018-4772-2

Islet preparation	1	2	3	4	5	6	7	8[a]
**MANDATORY INFORMATION**								
Unique identifier	27-7-17	2-8-17	21-8-17	R443	175/22			
Donor age (years)	64	74	74	65	54			
Donor sex (M/F)	F	M	M	F	M			
Donor BMI (kg/m^2)	22.2	26	22.5	23	27.7			
Donor HbA_{1c} or other measure of blood glucose control	N/A	N/A	N/A	5.1	N/A			
Origin/source of islets[b]	Pisa, Italy	Pisa, Italy	Pisa, Italy	Edmonton, Canada	Barcelona, Spain			
Islet isolation centre	Department of Clinical and Experimental Medicine, University of Pisa, Pisa, Italy	Department of Clinical and Experimental Medicine, University of Pisa, Pisa, Italy	Department of Clinical and Experimental Medicine, University of Pisa, Pisa, Italy	Alberta Diabetes Institute Isletcore, Edmonton, Canada.	Hospital Universitari Bellvitge. University of Barcelona. Barcelona, Spain. Bellvitge Biomedical Research Institute (IDIBELL), Barcelona, Spain			
Donor history of diabetes? Please select yes/no from drop down list	No	No	No	No	No			

**If Yes, complete the next two lines if this information is available**								
Diabetes duration (years)								
Glucose-lowering therapy at time of death[c]								
**RECOMMENDED INFORMATION**								
Donor cause of death	Cerebral hemorrhage	Cerebral hemorrhage	Cerebral hemorrhage	NDD - Neurological	Anoxia due to multiple strokes			
Warm ischaemia time (h)								
Cold ischaemia time (h)								
Estimated purity (%)	42% beta cells	46 % beta cells	31 % beta cells	65% beta cells	57% beta cells			
Estimated viability (%)	92% viable cells	82 % viable cells	89% viable cells	68% viable cells	65% viable cells			
Total culture time (h)[d]								
Glucose-stimulated insulin secretion or other functional measurement[e]								
Handpicked to purity? Please select yes/no from drop down list	Yes	Yes	Yes	Yes	Yes			
Additional notes	Viability was assessed by Hoechst 33342 and propidium iodide staining after dispersion and transfection with a control siRNA							

[a]If you have used more than eight islet preparations, please complete additional forms as necessary
[b]For example, IIDP, ECIT, Alberta IsletCore
[c]Please specify the therapy/therapies
[d]Time of islet culture at the isolation centre, during shipment and at the receiving laboratory
[e]Please specify the test and the results
N/A not available

ESM Fig.1

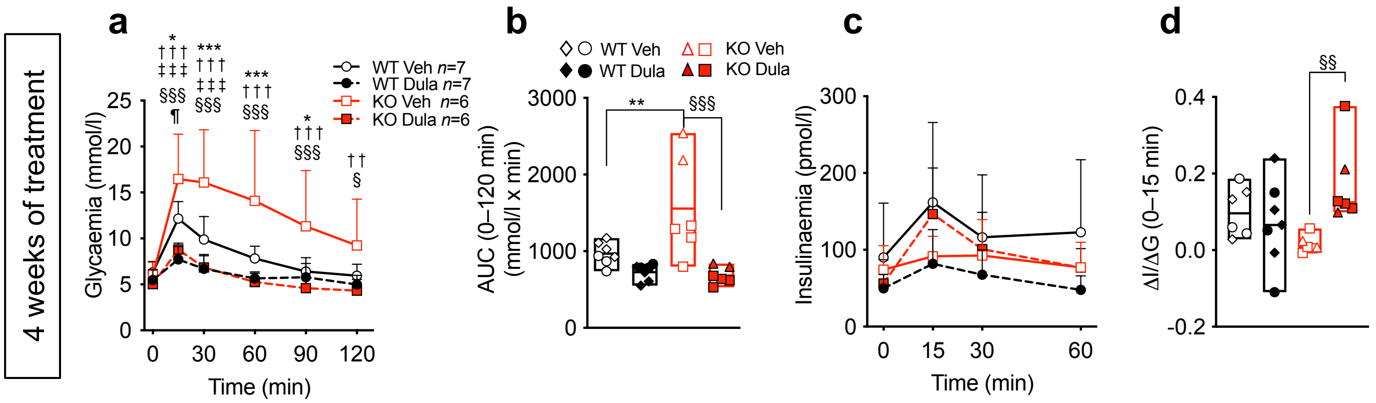

ESM Fig. 1. Four weeks of dulaglutide treatment prevents glucose intolerance in Wfs1-deficient mice. (a-d) Metabolic data of *Wfs1* KO mice and WT littermates treated for 4 weeks with dulaglutide (1 mg/kg/4 days, Dula, *n*=6 per group) or saline solution (Veh, *n*=6-7 per group). (a) IPGTT glycaemia, (b) AUC and (c) insulinaemia during the IPGTT. (d) Insulinogenic index. Results are expressed as mean and SD. Circles and squares represent individual female mice, triangles and diamonds male mice. Extremities of floating bars are maximal and minimal values; horizontal line shows median. *p<0.05, **p<0.01, ***p<0.001 KO Veh vs WT Veh, †† p<0.01, ††† p<0.01 other time points vs time zero (T0) in KO; ‡‡‡ p<0.001 other time points vs T0 in WT, § p<0.05, §§p<0.01, §§§ p<0.001 Dula vs Veh in KO, ¶ p<0.05 Dula vs Veh in WT by 2-way or one-way ANOVA (as suitable) followed by Sidak's or Dunn's correction for multiple comparisons.

ESM Fig. 2

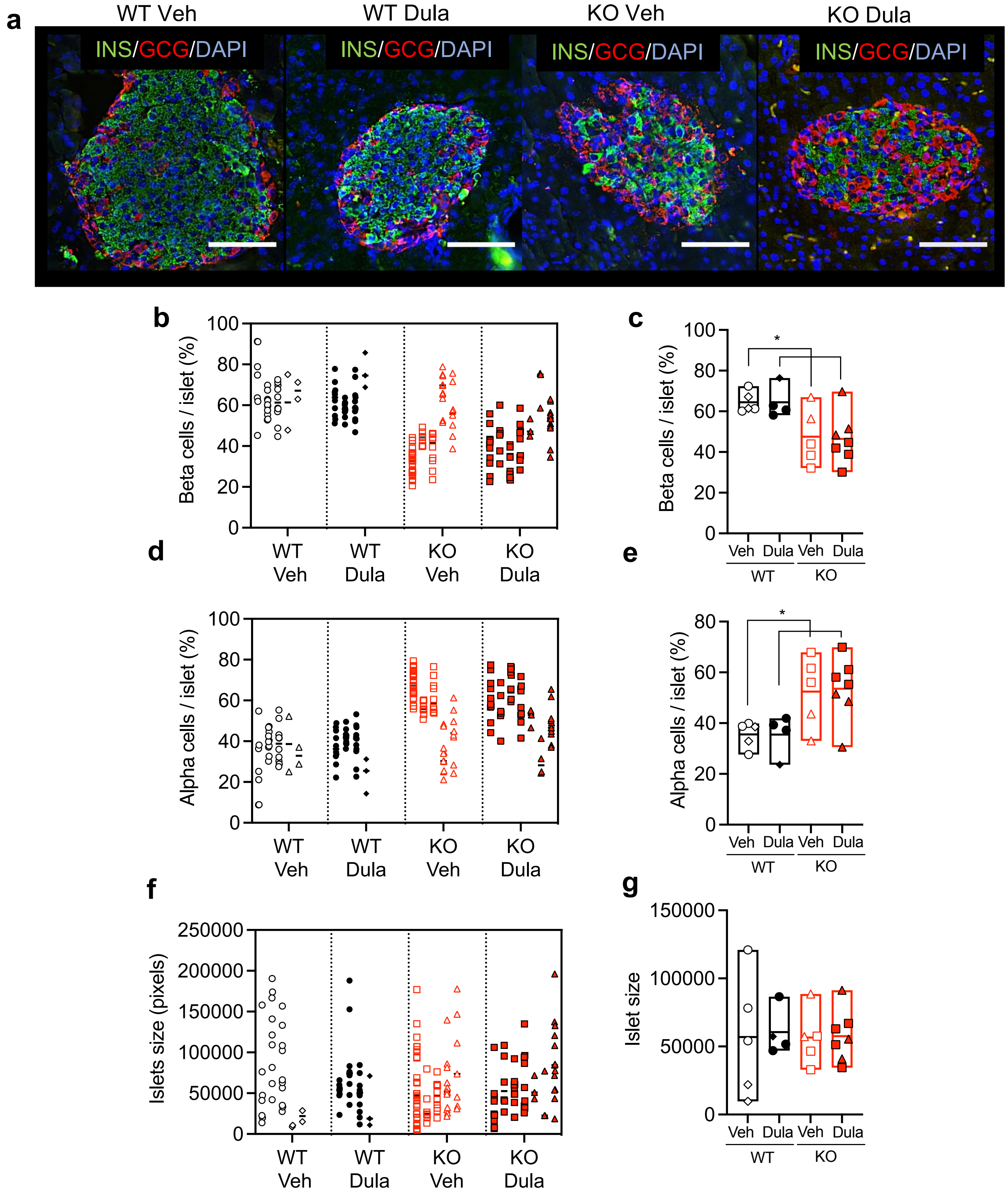

ESM Fig. 2. Dulaglutide treatment does not prevent beta cell loss in Wfs1 KO mice. At the end of the study described in Fig. 1, mice were killed, and pancreas of randomly selected animals fixed and processed for histological analysis. (a) Representative images of islets of WT or *Wfs1* KO mice injected with saline (Veh) or dulaglutide (Dula). Insulin is shown in green and glucagon in red; nuclei were stained with Dapi (blue), scale bar: 50 µm. (b-e) Quantification of beta and alpha cell proportion per islet (assessed by manual counting). (f-g) Islet size determined by Image J. (b, d and f) Each symbol represents an individual islet. Islets from the same mouse are stacked in columns. (c, e and g) Each point is average islet size, beta or alpha cell proportion per mouse. The extremities of the floating bars are maximal and minimal values and the horizontal line shows median. **p<0.01, Wfs1 KO vs WT by one way Anova with Šídák's correction for multiple comparisons.

ESM Fig. 3

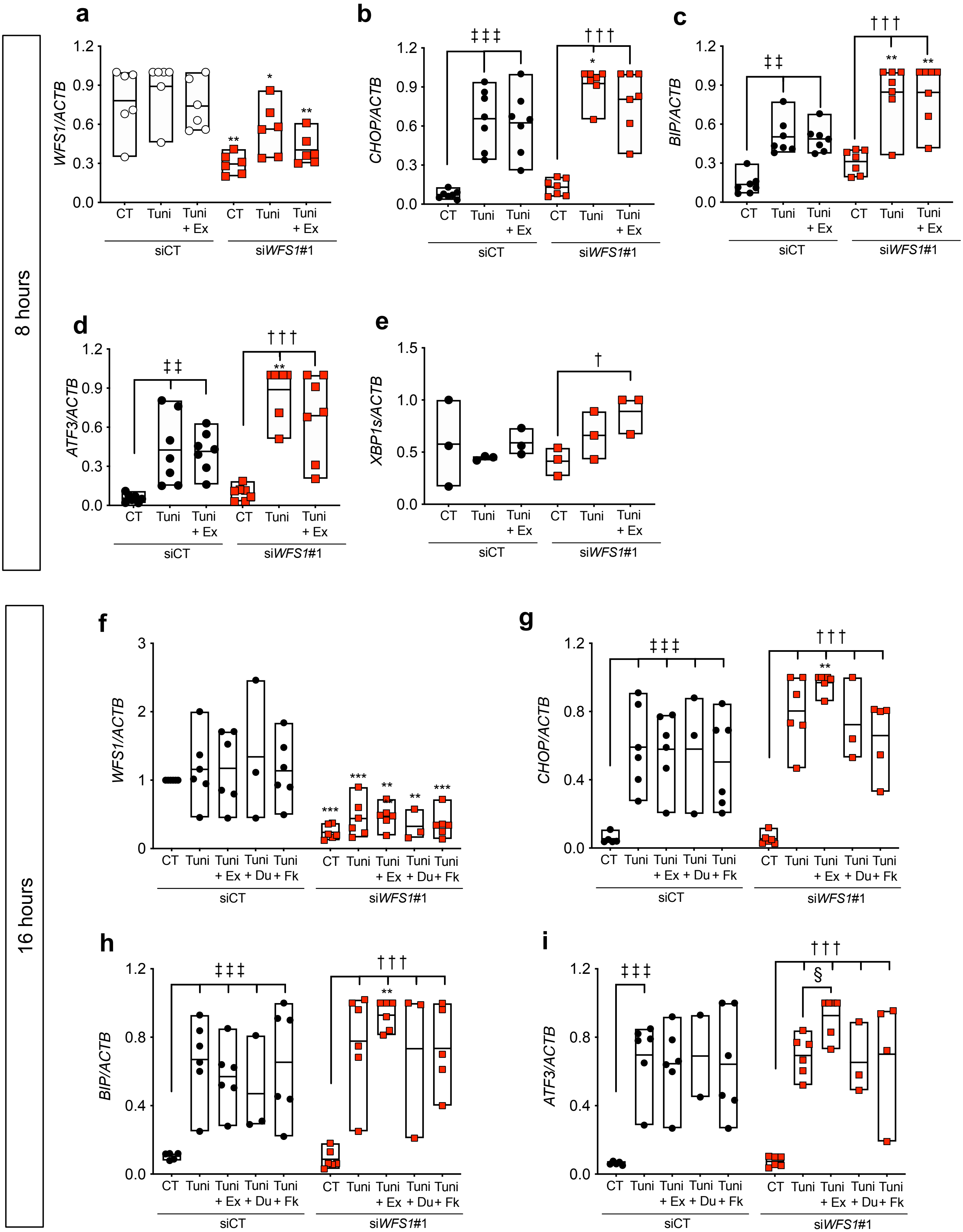
8 hours
16 hours
a
b
c
d
e
f
g
h
i
WFS1/ACTB
CHOP/ACTB
BIP/ACTB
ATF3/ACTB
XBP1s/ACTB
CT
Tuni
Tuni + Ex
Tuni + Du
Tuni + Fk
siCT
siWFS1#1

ESM Fig. 3. Exenatide does not alleviate ER stress in WFS1-silenced EndoC-βH1 cells. EndoC-βH1 cells were transfected with control siRNA (siCT) or siRNA targeting *WFS1* (si*WFS1*#1). 72h after transfection cells were exposed or not (CT) to the ER stressor tunicamycin (Tuni, 5 μg/ml) alone or in combination with 50 nmol/l exenatide (Ex), 50 nmol/l dulaglutide (Du) or 20 μmol/l forskolin (Fk) for 8h (a-e) or 16h (f-i). mRNA expression of *WFS1* and the ER stress markers *CHOP, BIP, ATF3 and XBP1s* was measured by real-time PCR, normalized to the reference gene *ACTB* and expressed as fold of the highest value in each experiment. Extremities of floating bars are maximal and minimal values; horizontal line shows median. Individual points represent independent experiments. **p*<0.05, ***p*<0.01, ****p*<0.001 si*WFS1* vs siCT; †*p*<0.05, †††*p*<0.001 treated vs CT in si*WFS1* cells; ‡‡*p*<0.01, ‡‡‡*p*<0.01 treated vs Ct in siCT cells; §*p*<0.05 Tuni + Ex vs Tuni by two-way ANOVA with Sidak's or Tukey's tests for multiple comparisons.

ESM Fig. 4

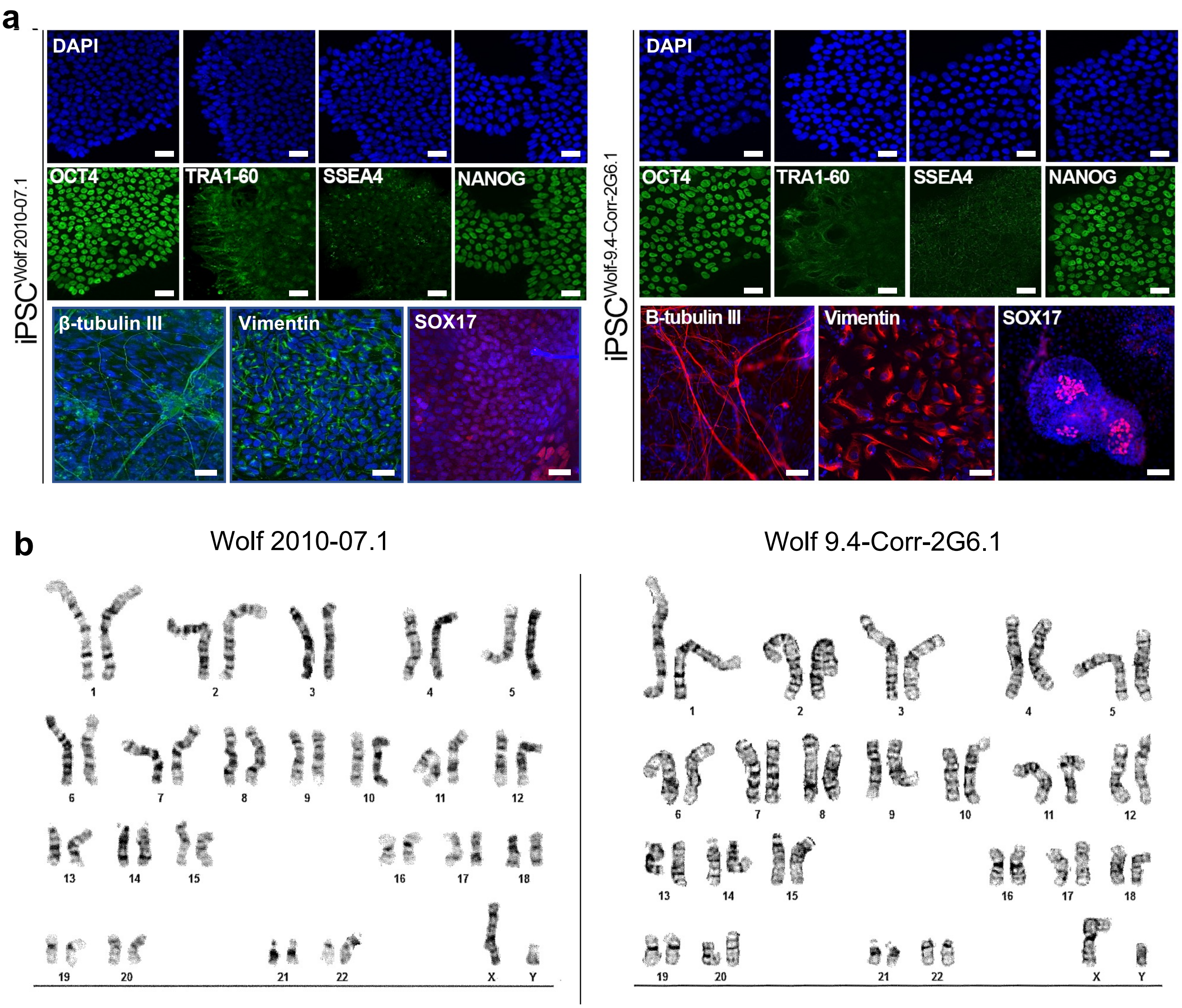

ESM Fig. 4. Characterization of the iPSC lines Wolf 2010-07.1 and Wolf 9.4-Corr-2G6.1 (a) Top panels, immunofluorescent staining of iPSCs for the pluripotency markers octamer binding protein 4 (OCT4), T cell receptor alpha locus (TRA1-60), stage-specific embryonic antigen 4 (SSEA4) and homeobox transcription factor nanog (NANOG). Lower panels, immunofluorescent staining for β-tubulin III, vimentin and transcrisption factor SOX-17 (SOX17) in iPSCs-derived embryoid bodies. In all pictures scale bars correspond to 10 µm. These proteins were used as markers of ectoderm, mesoderm, and endoderm, respectively. (b) iPSC karyotype of the two cell lines showing no chromosomal alterations.

ESM Fig. 5

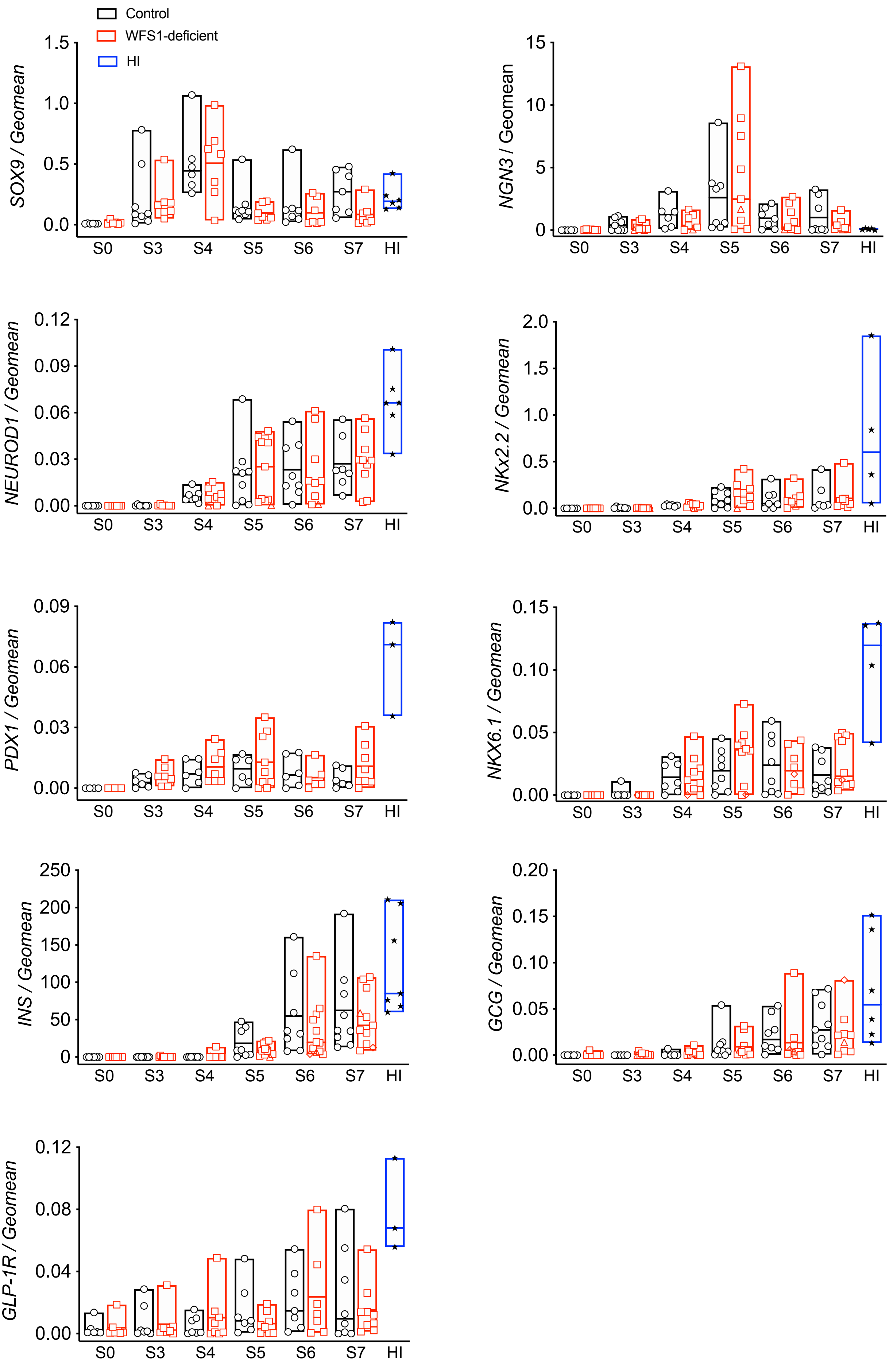

Control
WFS1-deficient
HI
SOX9 / Geomean
NGN3 / Geomean
NEUROD1 / Geomean
NKx2.2 / Geomean
PDX1 / Geomean
NKX6.1 / Geomean
INS / Geomean
GCG / Geomean
GLP-1R / Geomean
S0
S3
S4
S5
S6
S7
HI

ESM Fig. 5. Gene expression during differentiation of iPSCs into pancreatic beta cells. mRNA expression of the differentiation markers *SOX9*, neurogenin3 (*NGN3*), *NEUROD1, NKX2.2, PDX1, NKX6.1*, insulin (*INS*), glucagon (*GCG*) and *GLP-1R* at iPSC stage 0 (S0) and across differentiation stages 3 to 7 (S3 to S7) for isogenic control line Wolf-9.4-Corr-2G6.1 (Control, in black) and patient lines Wolf-2010-11.1, Wolf-2011-13.2, and Wolf-2010-9.4 (WFS1, in red). The blue bar shows mRNA expression of the same markers in human islets from adult organ donors (HI). Data were normalized to the geometric mean of reference genes *GAPDH* and *ACTB*. Individual data points represent independent samples. Extremities of floating bars are maximal and minimal values, horizontal line shows median.

ESM Fig. 6

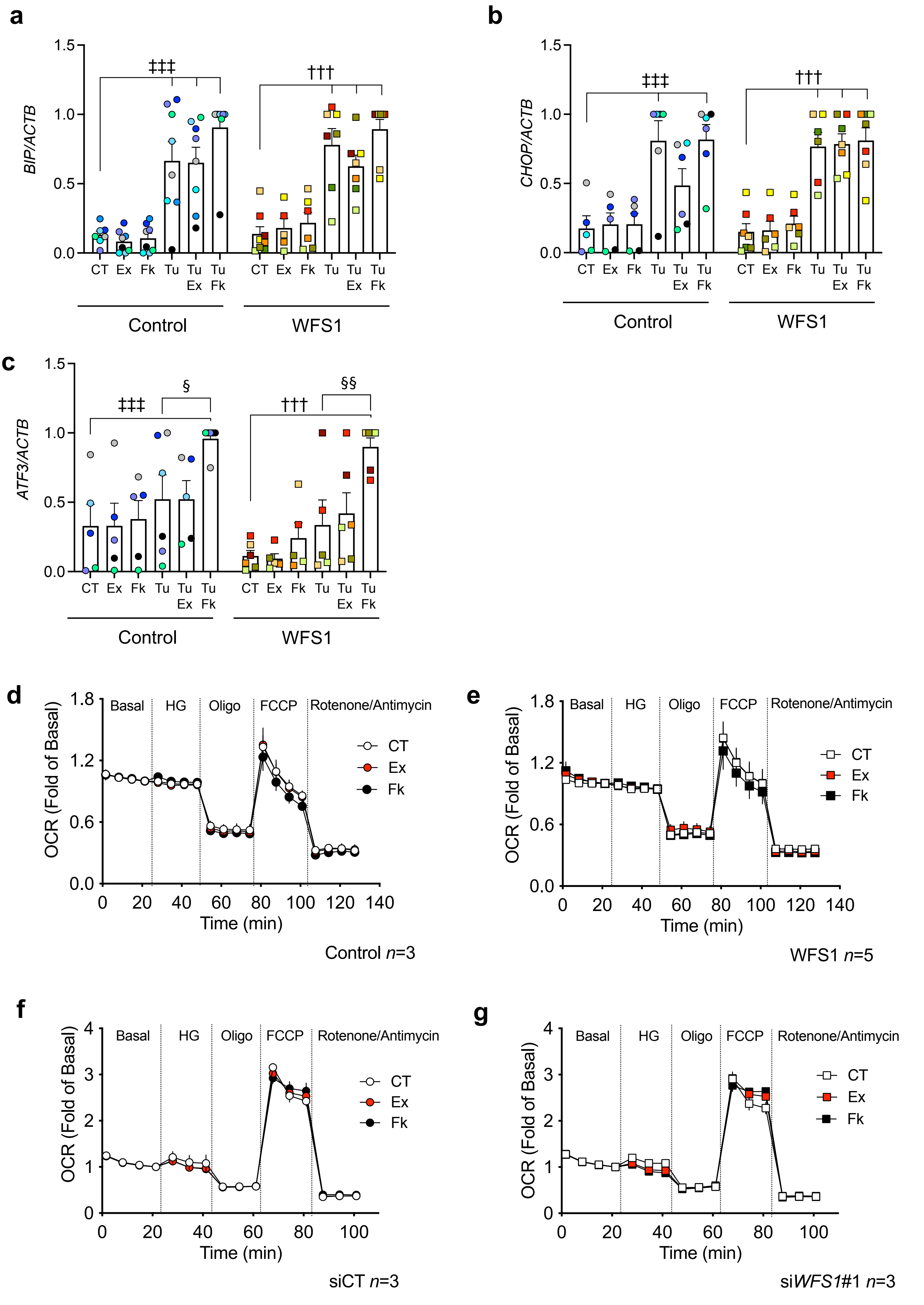
a
BIP/ACTB
‡‡‡
†††
CT
Ex
Fk
Tu
Tu Ex
Tu Fk
Control
WFS1
b
CHOP/ACTB
c
ATF3/ACTB
§
§§
d
e
f
g
Basal
HG
Oligo
FCCP
Rotenone/Antimycin
OCR (Fold of Basal)
Time (min)
Control n=3
WFS1 n=5
siCT n=3
siWFS1#1 n=3

ESM Fig. 6. ER stress markers and mitochondrial function in control and WFS1-deficient iPSC-derived beta cells and EndoC-βH1 cells. The WFS1-deficient iPSC line Wolf-2010-9.4 and its isogenic control Wolf-9.4-Corr-2G6.1 were differentiated into pancreatic beta cells. At the end of the differentiation, cells were cultured for 24h with or without exenatide (Ex) or forskolin (Fk), and then exposed or not (CT) for 48h to tunicamycin (Tu) alone or combined with exenatide or forskolin. (a-c) Expression of the ER stress markers *BIP, CHOP* and *ATF3* was assessed by real-time PCR, normalized to the reference gene *ACTB,* and expressed as fold of the highest value in each experiment. Individual data points represent independent iPSC differentiations, identified with a different color. Extremities of floating bars are maximal and minimal values; horizontal line shows median. (d-g) Mitochondrial function assessed by Seahorse in control and WFS1-deficient iPSC-derived dispersed beta cell aggregates (d-e), and control and WFS1-silenced EndoC-βH1 cells (f-g) exposed or not (CT) for 72h to exenatide or forskolin. Oxygen consumption rate (OCR) in isogenic control (d), Wolf-2010-9.4 (e), control EndoC-βH1 cells (f) and *WFS1*-silenced EndoC-βH1 cells (g), is expressed as fold change of the last basal reading in each sample, n=3-5 per group. ‡‡‡ $p<0.001$ treated vs CT in control cells; ††† $p<0.001$ treated vs CT in WFS1-deficient cells, §$p<0.05$, §§$p<0.01$ Tu vs Tu FK, by two-way ANOVA with Sidak’s or Tukey’s tests for multiple comparisons.

ESM Fig. 7

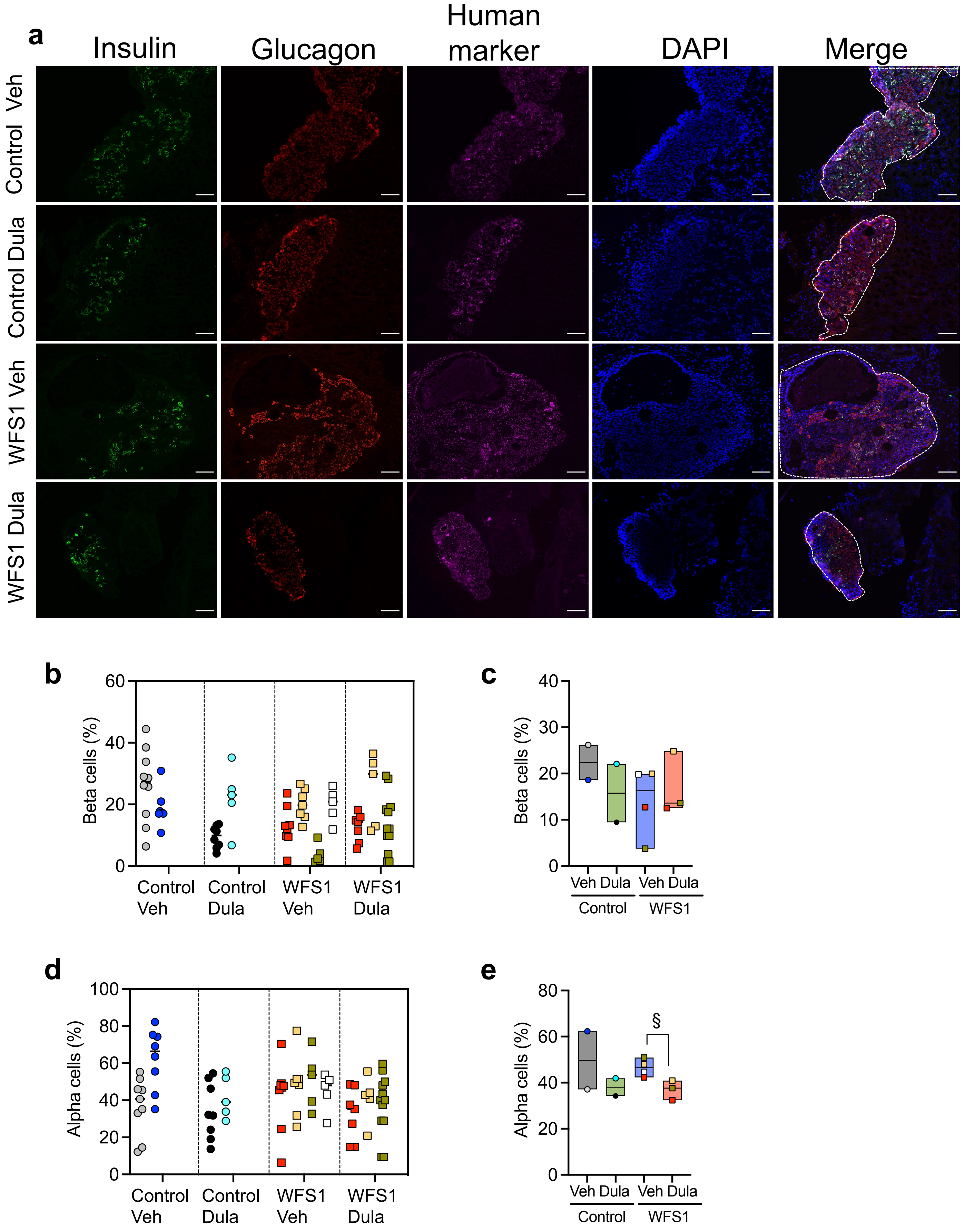
a
Insulin
Glucagon
Human marker
DAPI
Merge
Control Veh
Control Dula
WFS1 Veh
WFS1 Dula
b
Beta cells (%)
0
20
40
60
Control Veh
Control Dula
WFS1 Veh
WFS1 Dula
c
Beta cells (%)
0
10
20
30
40
Veh
Dula
Veh
Dula
Control
WFS1
d
Alpha cells (%)
0
20
40
60
80
100
Control Veh
Control Dula
WFS1 Veh
WFS1 Dula
e
Alpha cells (%)
0
20
40
60
80
§
Veh
Dula
Veh
Dula
Control
WFS1

ESM Fig. 7. Impact of dulaglutide on beta and alpha cell proportions in Wolfram syndrome iPSC-derived grafts. At the end of the experiment described in Fig. 6 and after kidney perifusion, grafts were fixed in formalin and the tissue used for histological analysis. (a) Representative immunofluorescence images of control and WFS1 iPSC-derived grafts retrieved from mice injected for 12 weeks with saline (Veh) or dulaglutide (Dula). Insulin staining is shown in green and glucagon in red; human nuclei in pink (used to differentiate human graft from mouse tissue), and nuclei in blue (Dapi). Scale bars 50 µm. (b-e) Quantification of beta and alpha cell proportion in the graft (assessed by manual counting). (b and d) Each symbol represents the beta and alpha cell proportion in one picture. Values from pictures from the same mouse are stacked in columns. (c and e) Average beta or alpha cell proportion in each graft. Symbols with a same color in two different mice indicate that these animals were transplanted with aggregates from the same differentiation. Extremities of floating bars are maximal and minimal values; horizontal line shows median. § Dula vs Veh by paired ttest.
